# Effects of first-generation in utero exposure to diesel engine exhaust on second-generation placental function, fatty acid profiles and foetal metabolism in rabbits: preliminary results

**DOI:** 10.1038/s41598-019-46130-x

**Published:** 2019-07-04

**Authors:** Delphine Rousseau-Ralliard, Sarah A. Valentino, Marie-Christine Aubrière, Michèle Dahirel, Marie-Sylvie Lallemand, Catherine Archilla, Luc Jouneau, Natalie Fournier, Christophe Richard, Josiane Aioun, Anaïs Vitorino Carvalho, Lecardonnel Jérôme, Rémy Slama, Véronique Duranthon, Flemming R. Cassee, Pascale Chavatte-Palmer, Anne Couturier-Tarrade

**Affiliations:** 1 UMR BDR, INRA, ENVA, Université Paris Saclay, Jouy en Josas, France; 2 PremUp Foundation, Paris, France; 3 University Paris-Sud, EA 4041/4529 Lip (Sys)2, UFR de Pharmacie, Châtenay-Malabry, France; 4 Hôpital Européen Georges Pompidou (AP-HP), Laboratoire de Biochimie, UF Cardio-Vasculaire, Paris, France; 5 GABI CRB GADIE, INRA, Université Paris Saclay, Jouy en Josas, France; 6 Inserm, Univ. Grenoble Alpes, CNRS, IAB joint Research Center, Team of Environmental Epidemiology Applied to Reproduction and Respiratory Health, Grenoble, France; 7 Centre for Sustainability, Environment and Health, National Institute for Public Health and the Environment, Bilthoven, Netherlands; 8 Institute of Risk Assessment Sciences, Utrecht University, Utrecht, Netherlands

**Keywords:** Fatty acids, Developmental biology

## Abstract

Atmospheric pollution has major health effects on directly exposed subjects but intergenerational consequences are poorly characterized. We previously reported that diesel engine exhaust (DE) could lead to structural changes in the placenta of *in utero* exposed rabbits (first generation, F1). The effects of maternal exposure to DE were further studied on second-generation (F2) rabbits. Pregnant F0 females were exposed to filtered, diluted DE (1 mg/m^3^, median particle diameter: 69 nm) or clean filtered air (controls) for 2 h/day, 5 days/week by nose-only exposure during days 3–27 post-conception (dpc). Adult female offspring (F1) were mated to control males: F1 tissues and F2 foeto-placental units were collected at 28 dpc and placental structure and gene expression (microarray) analysed. Fatty acid profiles were determined in foetal and maternal plasma, maternal liver and placenta. In F1, compared to controls, hepatic neutral lipid contents were increased in exposed animals without change in the blood biochemistry. In F2, the placental lipid contents were higher, with higher monounsaturated fatty acids and reduced pro-inflammatory arachidonic acid (AA), without placental structural changes. Conversely, the proportion of anti-inflammatory n-3 polyunsaturated fatty acids in F2 plasma was increased while that of AA was decreased. Gene set enrichment analyses (GSEA) of F2 placenta transcriptomic data identified that the proteasome complex and ubiquitin pathways genes were over-represented and ion channel function and inflammation pathways genes were under-represented in exposed animals. These preliminary results demonstrate that diesel engine exhaust exposure and *in utero* indirect exposure should be considered as a programming factor within the context of the DOHaD (Developmental Origins of Health and Disease) with a probable intergenerational transmission.

## Introduction

Air pollution has become a major health concern in most industrialized and non-industrialized countries over the last decades^1^. Diesel exhaust (DE) is a major contributor to air pollution worldwide. DE comprised a large variety of compounds, including a solid particular fraction that mainly consists of soot, and liquid and gaseous fractions^2^. Current European and North American regulation services limit the diesel motor vehicle emission of carbon monoxide, nitric oxides, hydrocarbons, and particles (size and concentration). Thus, modern motors are equipped with particle filters that reduce PM10 (particulate matter with an aerodynamic diameter below 10 µm) and PM2.5 (<2.5 μm) emissions. Ultrafine or nanoparticles (<100 nm), however, may not be efficiently filtered from the exhaust^3^ and their impact on human health needs to be evaluated. Several studies in humans and animals have associated reduced foetal growth, low birth weight and long-term offspring outcomes with *in utero* exposure to air pollutants, such as DE^4–10^. Several studies have indicated that placental function is affected by air pollutants^11–13^, probably through inflammation and oxidative stress. Comparisons between studies, however, remain difficult due to the large array of exposition routes and windows of exposure as well as the pollutant and particle concentrations used.

The maternal environment, extended across the full range of maternal conditions, is an important component of an offspring’s risk of developing metabolic disease in adulthood. These maternal effects are based on the epigenetic memory of early developmental conditions and there is some evidence for their transmission to succeeding generations^14,15^.

Experimental studies of the intergenerational effects of exposure to atmospheric pollution have mostly been conducted in rodents, and mainly focused on offspring reproductive function. Thus, in rodents, maternal (F0) exposure to DE or nanoparticles was shown to reduce daily sperm production and alter steroidogenesis in first generation (F1) males^16^. In females, the ovarian reserve was reported to be decreased (reduced number of primary follicles) after maternal exposure to DE^17^. These results suggest that offspring gonads and gametes may be affected, thus possibly inducing second-generation effects, as described with other environmental factors^18,19^. In any case, little is known about intergenerational effects of DE exposure. One Brazilian study showed that in mice, continuous exposure to urban San Paulo pollution over 3 generations altered second generation female fertility and increased the incidence of foetal abortion, decreased foetal weight and altered placental structure in the third generation^8,9^.

Here, the intergenerational effects of *in utero* F1 exposure to DE on the second (F2) generation were studied using a rabbit model. The rabbit was chosen as a particularly appropriate model because compared to rodent placenta the haemochorial placental structure of a rabbit is closer to that of a human^20,21^. The data presented here build upon previously published data generated in the same experiment, demonstrating that F2 foeto-placental biometry was not disturbed consecutively to the *in utero* exposure of the F1 generation to DE^7^. Nevertheless, total plasma cholesterol and non-HDL cholesterol were reduced (−25.9% and −26.0%, respectively) and plasma triglycerides were increased (+25.9%) in the macroscopically normal F2 foetuses of the exposed group compared to those of the control group^7^.

In the present study, F1 adult end-gestational metabolism (with a focus on liver) and placental function in the second (F2) generation were explored at the biochemical, histological and molecular levels to understand how maternal metabolism through the placenta contributed to foetal dyslipidaemia without affecting biometry.

## Results

In this study, hereafter, CC refers to F2 foeto-placental units collected from F1 control (C) dams, and EC refers to F2 foeto-placental units collected from *in utero*-exposed F1 (E) dams, with no further exposure of these F1 females after birth until adulthood and during their own gestation. In the previously published part of this study by Valentino *et al*., 2016, although F2 placental and foetal biometry were not different between CC and EC gestations, significant differences were found for F2 foetal plasma biochemistry as described above in the Introduction^7^.

### Maternal biochemistry and histological analyses of the liver at 28 dpc

In contrast with the findings in the foetuses, there was no significant difference for any studied plasma biochemical parameter in the F1 adult dams at 28 dpc (Supplementary Table S1). Nevertheless, using a histological approach, liver steatosis was observed in exposed compared to control dams (Supplementary Fig. S1). The hepatocytes in pregnant exposed F1 adult rabbits at 28 dpc presented numerous intracellular micro and macro vacuoles, corresponding to lipid vesicles, which were not positive for Periodic Acid Schiff (PAS, glycogen staining), compared to those in pregnant control F1 females.

### Placental stereology

No change in F2 foetal or placental weight was reported previously^7^. As a reminded, the litter sizes ranged from 6 to 13 fetuses without difference in sex ratio or change in fetal weight between groups. The cellular composition of the labyrinthine area was explored by stereology (Supplementary Fig. S2) and there was no significant difference between the two groups for the relative volume fraction and surface density of the maternal blood space, trophoblast and foetal vessels (Supplementary Table S2).

### Fatty acid analyses: fatty acid concentrations

Maternal (F1) and foetal (F2) plasma fatty acid concentrations and maternal liver and placenta tissue FA concentrations were determined (Table 1). Phospholipids (PL, corresponding to cell membranes) and neutral lipids (NL, corresponding to intracellular lipid storage) were determined in the placentas and maternal livers. In the placentas, the intracellular neutral lipid concentration was significantly increased in EC compared to that in CC (+95.1%, p = 0.0002) whereas there was no difference between the groups for phospholipid fatty acid concentrations.

**Table 1:** Fatty acid concentrations in plasma and liver from control (C) or exposed (E) F1 dams and in F2 fetal plasma and placentas (CC and EC, respectively) at 28 dpc.

Maternal tissues	Effective		Median [Q1; Q3]		Fully adjusted linear model		
	C	E	Control	Exposed	β value	CI	p-value
Maternal F1 plasma (µg/mL)	3	9	396.4 [325.4; 463.6]	390.2 [359.5; 454.0]	7.03	[−98.21; 112.26]	0.901
Maternal F1 liver (NL) (mg/g of liver)	3	9	44.7 [41.1; 65.4]	107.6 [89.9; 125.5]	56.37	[16.12; 96.61]	0.050*
Maternal F1 liver (PL) (mg/g of liver)	3	9	18.8 [14.1; 21.2]	18.5 [15.6; 23.4]	−3.43	[−10.49; 3.64]	0.391
**Fetal tissues**	**CC**	**EC**	**CC**	**EC**	**β value**	**CI**	**p-value**
F2 Placenta (NL) (mg/g of placenta)	11	11	1.7 [1.5; 1.9]	3.4 [2.8; 4.2]	1.74	[1.26; 2.22]	0.0002***
F2 Placenta (PL) (mg/g of placenta)	12	12	3.3 [2.9; 3.7]	5.4 [3.4; 6.4]	2.03	[−0.16; 4.22]	0.109
F2 Fetal plasma (µg/mL)	12	12	385.4 [346.7; 407.9]	482.7 [460.9; 577.8]	113.84	[41.58; 186.09]	0.021*

*Data are expressed as Median[Q1;Q3]. C and CC: F1 and F2 control samples, respectively; CI: 95% Confidence Interval; E and EC: F1 and F2 exposed samples, respectively; NL: neutral lipids for intracellular lipid storage; PL: phospholipids for cell membranes* “Statistics with *P < 0.05” and **P < 0.01.

Because EC placentas were enriched in fats, the fatty acid profiles of F1 maternal and F2 foetal plasma were analysed. The fatty acid concentrations in foetal plasma were significantly increased (+25.2%, p = 0.021) in EC foetuses compared to those of CC. Maternal plasma fatty acid concentrations were similar between groups, confirming the biochemical observations. Because maternal plasma fatty acid concentrations were not different among groups, the maternal liver was further explored to understand the increase in the fatty acid content in both foetal plasma and placenta. Thus, intracellular neutral lipid concentrations were significantly increased in the livers of exposed dams compared to those of control dams (p = 0.049), confirming the histological observations of liver steatosis in exposed F1 females once pregnant.

### Qualitative fatty acid analyses: fatty acid profiles

#### Fatty acid profile in F1 maternal plasma

The proportion of palmitic acid (PA, C16:0) tended to be higher (+9.3%, p = 0.067) whereas C16:1n-7 and C18:3n-3 were decreased (−39.1%, p = 0.027 and −36.7%, p = 0.019, respectively) in the maternal plasma of exposed F1 dams compared to that in the controls (Table 2). Polyunsaturated fatty acids (PUFA), especially those from n-3 series, tended to be reduced in exposed compared to control dams (−27.8%, p = 0.068). PCA confirmed these differences but no specific fatty acid signature related to their *in utero* exposure could be identified (Supplementary Fig. S3).

**Table 2:** Fatty acid profiles in plasma of control (C) and *in utero* exposed (E) F1 adult pregnant dams at 28dpc (% of total fatty acids).

Variable	Number of dams		Median [Q1; Q3]		Fully adjusted linear model		
	C	E	Control	Exposed	β value	CI	p-value
C16:0 (PA)	3	9	29.1 [28.5; 30.1]	31.8 [30.4; 32.8]	2.55	[0.53; 4.57]	0.067
C16:1n-7	3	9	10.4 [8.2; 11.5]	6.4 [6.1; 8.2]	−3.15	[−4.98; −1.31]	0.027*
Total C16:1	3	9	10.9 [8.6; 11.9]	7.1 [6.6; 8.6]	−3.10	[−4.91; −1.28]	0.028*
C18:0 (SA)	3	9	8.0 [6.0; 9.2]	10.0 [8.7; 12.7]	3.73	[−0.90; 8.36]	0.186
Total C18:1	3	9	25.2 [25.0; 28.8]	25.4 [24.2; 29.5]	−1.03	[−8.21; 6.15]	0.791
C18:2 n-6 (LA)	3	9	17.7 [16.9; 17.9]	16.2 [15.4; 17.3]	−1.25	[−2.44; −0.06]	0.106
C18:3 n-3 (ALA)	3	9	2.8 [2.5; 3.3]	1.8 [1. 6; 2.0]	−1.22	[−1.85; −0.59]	0.019*
C20:4 n-6 (AA)	3	9	1.5 [0.9; 1.6]	1.0 [0.8; 1.7]	−0.06	[−0.84; 0.72]	0.892
C20:5 n-3 (EPA)	3	9	0.02 [0.00; 0.04]	0.04 [0.02; 0.37]	0.18	[−0.34; 0.71]	0.529
C22:4 n-6	3	9	0.04 [0.04; 0.11]	0.04 [0.03; 0.06]	−0.01	[−0.08; 0.06]	0.794
C22:5 n-6	3	9	0.08 [0.00; 0.08]	0.06 [0.05; 0.11]	0.04	[−0.05; 0.14]	0.397
C22:5 n-3 (DPA)	3	9	0.07 [0.05; 0.08]	0.05 [0.02; 0.08]	−0.01	[−0.07; 0.05]	0.710
C22:6 n-3 (DHA)	3	9	0.05 [0.04; 0.07]	0.04 [0.03; 0.06]	−0.01	[−0.05; 0.03]	0.608
Total SFA	3	9	39.9 [38.7; 40.0]	44.9 [41.5; 47.5]	6.37	[−0.98; 13.71]	0.162
Total MUFA	3	9	37.8 [36.7; 37.9]	34.6 [31.4; 37.3]	−4.04	[−10.34; 2.25]	0.272
Total n-6 PUFA	3	9	19.7 [19.4; 20.1]	18.9 [17.6; 19.3]	−1.16	[−3.05; 0.72]	0.289
Total n-3 PUFA	3	9	3.2 [2.9; 3.7]	2.3 [2.1; 2.8]	−1.11	[−2.01; −0.22]	0.068
n-6/n-3 PUFA ratio	3	9	6.4 [5.3; 6.8]	8.2 [6.6; 9.5]	2.64	[−0.59; 5.88]	0.181
PUFA/SFA ratio	3	9	0.58 [0.56; 0.60]	0.50 [0.42; 0.52]	−0.11	[−0.19; −0.03]	0.049*
DNL index	3	9	1.7 [1.6; 1.7]	1.9 [1.8; 2.1]	0.30	[0.06; 0.53]	0.070

*Data are expressed as Median[Q1;Q3], in % of total FA. AA: C20:4n-6, arachidonic acid; ALA: C18:3n-3, alpha-linolenic acid; CI: Confidence Interval; DHA: C22:6n-3, docosahexaenoic acid; DNL index: de novo lipogenesis (C16:0/C18:2n-6 ratio)*^22,23^*; DPA: C22:5n-3, docosapentaenoic acid; EPA: C20:5n-3, Eicosapentaenoic acid; LA: C18:2n-6, linoleic acid; MUFA: monounsaturated fatty acids; PA: C16:0, palmitic acid; PL: phospholipids; PUFA: polyunsaturated fatty acids; SA: C18:0, stearic acid; SFA: saturated fatty acids* “Statistics with *P < 0.05” and **P < 0.01.

#### Fatty acid profile in F2 placental membranes (phospholipids, PL)

Compared to controls, EC placental membranes (PL) (Table 3) were characterized by lower C15:0 and SFA (−14.4%, p = 0.025 and −1.6%, p = 0.024, respectively) contents, as well as lower long chain polyunsaturated fatty acids (LC n-6 PUFA, ≥20 carbons), especially C20:2n-6 and C22:4n-6 (−16.1%, p = 0.017 and −28.4%, p = 0.004) and LC n-3 PUFA, particularly docosapentaenoic acid (DPA, C22:5n-3, −8%, p = 0.031). The placental membranes were also characterized by higher monounsaturated fatty acid (MUFA) contents, especially C16:1n-9 (+21%, p = 0.02) compared to the placental membranes of controls. PCA performed on placental phospholipid fatty acid profiles confirmed these differences. A specific fatty acid signature enabling the discrimination of each group in placental membranes was identified (Supplementary Fig. S4), with the main plan representing 43.8% of the inertia of the data summarized in Table 3.

**Table 3:** Profile in fatty acid in F2 of control (CC) and exposed (EC) placenta phospholipids (PL) at 28 dpc (% of total fatty acids).

Variable	Number of placenta		Median [Q1; Q3]		Fully adjusted linear model		
	CC	EC	CC	EC	β value	CI	p-value
C15:0	12	12	0.52 [0.51; 0.56]	0.45 [0.42; 0.49]	−0.09	[−0.148; −0.027]	0.025*
C16:0 (PA)	12	12	29.3 [28.9; 30.0]	29.1 [28.9; 29.4]	−0.50	[−1.514; 0.51]	0.359
C16:1n-9	12	12	4.0 [3.6; 4.6]	4.9 [4.5; 5.0]	0.68	[0.234; 1.131]	0.020*
Total C16:1	12	12	6.1 [5.5; 6.7]	7.0 [6.7; 7.2]	0.83	[0.35; 1.307]	0.011*
C18:0 (SA)	12	12	17.3 [17.0; 17.6]	17.0 [16.5; 17.3]	−0.32	[−0.728; 0.083]	0.160
Total C18:1	12	12	19.3 [18.9; 19.7]	19.4 [18.8; 20.2]	0.38	[−0.663; 1.414]	0.497
C18:2 n-6 (LA)	12	12	11.8 [10.9; 12.1]	12.4 [11.8; 13.0]	0.63	[−0.051; 1.304]	0.110
C18:3n-6 (GLA)	12	12	0.58 [0.50; 0.69]	0.79 [0.75; 0.87]	0.20	[0.065; 0.328]	0.021*
C18:3n-3 (ALA)	12	12	0.09 [0.07; 0.09]	0.08 [0.07; 0.08]	−0.01	[−0.016; 0.002]	0.190
C20:2n-6	12	12	0.16 [0.14; 0.17]	0.13 [0.12; 0.14]	−0.03	[−0.048; −0.011]	0.017*
C20:3n-6 (DGLA)	12	12	5.2 [4.9; 5.6)	4.8 [4.6; 5.1]	−0.45	[−1.144; 0.238]	0.236
C20:4n-6 (AA)	12	12	2.9 [2.8; 3.1]	2.5 [2.4; 2.9]	−0.19	[−0.444; 0.062]	0.186
C20:5n-3 (EPA)	12	12	0.04 [0.03; 0.05]	0.04 [0.04; 0.04]	0.002	[−0.009; 0.012]	0.765
C22:4n-6	12	12	0.74 [0.67; 0.85]	0.53 [0.49; 0.62]	−0.21	[−0.313; −0.112]	0.004**
C22:5n-6	12	12	0.86 [0.80; 1.02]	0.70 [0.63; 0.90]	−0.17	[−0.417; 0.069]	0.200
C22:5n-3 (DPA)	12	12	0.69 [0.65; 0.78]	0.64 [0.50; 0.65]	−0.13	[−0.233; −0.035]	0.031*
C22:6n-3 (DHA)	12	12	0.26 [0.22; 0.27]	0.27 [0.22; 0.32]	0.01	[−0.054; 0.078]	0.732
Total SFA	12	12	49.3 [48.8; 49.8]	48.5 [47.8; 49.0]	−0.77	[−1.308; −0.241]	0.024*
Total MUFA	12	12	27.3 [26.2; 27.7]	28.2 [27.5; 29.3]	1.33	[0.165; 2.491]	0.058
Total n-6 PUFA	12	12	22.2 [27.8; 23.0]	22.1 [21.3; 23.1]	−0.64	[−1.814; 0.541]	0.320
Total n-3 PUFA	12	12	1.2 [1.1; 1.2]	1.1 [0.9; 1.2]	−0.13	[−0.279; 0.019]	0.127
n-6/n-3 PUFA ratio	12	12	19.2 [18.1; 19.7]	19.1 [18.6; 24.2]	1.88	[−1.714; 5.464]	0.335
PUFA/SFA ratio	12	12	0.47 [0.46; 0.49]	0.48 [0.47; 0.49]	−0.004	[−0.02; 0.012]	0.653

*Data are expressed as Median[Q1;Q3], in % of total FA. AA: C20:4n-6, arachidonic acid; ALA: C18:3n-3, alpha-linolenic acid; CI: Confidence Interval; DGLA: C20:3n-6, dihomo-gamma-linolenic acid; DHA: C22:6n-3, docosahexaenoic acid; DPA: C22:5n-3, docosapentaenoic acid; EPA: C20:5n-3, Eicosapentaenoic acid; GLA: C18:3n-6, gamma-linolenic acid; LA: C18:2n-6, linoleic acid; MUFA: monounsaturated fatty acids; PA: C16:0, palmitic acid; PL: phospholipids; PUFA: polyunsaturated fatty acids; SA: C18:0, stearic acid; SFA: saturated fatty acids* “Statistics with *P < 0.05” and **P < 0.01.

#### Fatty acid profile in F2 placental intracellular lipid storages (neutral lipids, NL)

EC placental intracellular lipid stores (neutral lipids, NL) (Table 4) were characterized by lower C14:0 and C15:0 contents (−14.2%, p = 0.004 and −12.1%, p = 0.035), a tendency for lower di-homo-gamma-linolenic acid contents (DGLA, C20:3n-6, −17.5%, p = 0.064), as well as lower PUFA contents (−17.3%, p = 0.014), especially arachidonic acid content (AA, C20:4n-6, −31.7%, p = 0.026); they were also characterized by higher MUFA (+12.7%, p = 0.02), particularly oleic acid (OA, C18:1n-9, +17.5%, p = 0.035), compared to the intracellular lipid stores of control placentas. PCA was performed on the fatty acid profiles of placental neutral lipids. A specific signature enabling discrimination between the two groups was identified, with the main plan representing 50.7% of the inertia of the data (Supplementary Fig. S5).

**Table 4:** Profile in fatty acid in F2 of control (CC) and exposed (EC) placenta NL at 28dpc (% of total fatty acids).

Variable	Number of placenta		Median [Q1; Q3]		Fully adjusted linear model		
	CC	EC	CC	EC	β value	CI	p-value
C14:0	11	11	2.2 [2.1; 2.4]	1.9 [1.8; 1.9]	−0.35	[−0.51; −0.19]	0.004**
C15:0	11	11	0.91 [0.84; 0.94]	0.80 [0.69; 0.85]	−0.11	[−0.19; −0.03]	0.035*
C16:0 (PA)	11	11	33.3 [32.4; 34.5]	34.0 [32.0; 34.8]	0.36	[−1.51; 2.23]	0.715
Total C16:1	11	11	7.5 [7.0; 7.8]	7.8 [7.4; 8.0]	0.81	[−0.48; 2.10]	0.253
C18:0 (SA)	11	11	9.6 [9.2; 10.1]	9.1 [8.7; 9.9]	−0.38	[−0.99; 0.23]	0.253
C18:1n-7	11	11	3.1 [3.0; 3.3]	3.2 [3.2; 3.4]	0.27	[0.06; 0.48]	0.043*
C18:1n-9 (OA)	11	11	22.6 [22.4; 24.7]	26.6 [24.1; 27.4]	3.06	[0.74; 5.37]	0.035*
Total C18:1	11	11	25.8 [25.4; 27.7]	29.8 [27.4; 30.8]	3.18	[1.01; 5.35]	0.023*
C18:2n-6 (LA)	11	11	8.9 [8.6; 9.5]	7.9 [6.7; 8.5]	−1.62	[−2.76; −0.48]	0.026*
C18:3n-3 (ALA)	11	11	0.49 [0.46; 0.51]	0.43 [0.36; 0.47]	−0.07	[−0.14; −0.01]	0.057
C20:3n-6 (DGLA)	11	11	5.0 [4.1; 6.3]	4.1 [3.7; 4.6]	−1.04	[−1.97; −0.10]	0.064
C20:4n-6 (AA)	11	11	1.6 [1.4; 1.7]	1.1 [1.0; 1.3]	−0.27	[−0.46; −0.09]	0.026*
C20:5n-3 (EPA)	11	11	0.09 [0.08; 0.10]	0.07 [0.06; 0.08]	−0.02	[−0.04; −0.01]	0.008**
C22:2n-6	11	11	0.17 [0.14; 0.21]	0.13 [0.09; 0.14]	−0.05	[−0.09; −0.01]	0.047*
C22:4n-6	11	11	0.54 [0.38; 0.65]	0.28 [0.26; 0.42]	−0.09	[−0.21; 0.03]	0.168
C22:5n-6	11	11	0.17 [0.14; 0.21]	0.12 [0.11; 0.18]	−0.04	[−0.09; 0.02]	0.256
C22:5n-3 (DPA)	11	11	0.22 [0.18; 0.24]	0.16 [0.12; 0.23]	0.01	[−0.04; 0.07]	0.643
C22:6n-3 (DHA)	11	11	0.04 [0.03; 0.09]	0.04 [0.03; 0.05]	−0.03	[−0.06; 0.002]	0.104
Total SFA	11	11	46.3 [44.8; 47.6]	45.5 [43.7; 47.0]	−0.03	[−2.48; 2.41]	0.978
Total MUFA	11	11	33.9 [33.3; 35.9]	38.16 [36.0; 38.9]	4.02	[1.35; 6.68]	0.02*
Total n-6 PUFA	11	11	18.0 [16.4; 19.4]	14.8 [14.4; 16.2]	−3.48	[−5.55; −1.41]	0.013*
Total n-3 PUFA	11	11	1.8 [1.3; 1.9]	1.7 [1.5; 1.8]	−0.02	[−0.22; 0.18]	0.876
n-6/n-3 PUFA ratio	11	11	10.5 [9.5; 13.6]	9.0 [8.3; 10.0]	−2.07	[−4.12; −0.03]	0.085
PUFA/SFA ratio	11	11	0.45 [0.38; 0.46]	0.35 [0.33; 0.41]	−0.07	[−0.13; −0.02]	0.039*

*Data are expressed as Median[Q1;Q3], in % of total FA. AA: C20:4n-6, arachidonic acid; ALA: C18:3n-3, alpha-linolenic acid; CI: Confidence Interval; DGLA: C20:3n-6, dihomo-gamma-linolenic acid; DHA: C22:6n-3, docosahexaenoic acid; DPA: C22:5n-3, docosapentaenoic acid; EPA: C20:5n-3, Eicosapentaenoic acid; LA: C18:2n-6, linoleic acid; MUFA: monounsaturated fatty acids; OA: C18:1n-9, oleic acid; PA: C16:0, palmitic acid; PL: phospholipids; PUFA: polyunsaturated fatty acids; SA: C18:0, stearic acid; SFA: saturated fatty acids* “Statistics with *P < 0.05” and **P < 0.01.

#### Fatty acid profile of F2 foetal plasma

Compared to controls, F2 EC foetuses (Table 5) were characterized by a tendency towards lower linoleic acid (LA, C18:2n-6, −18.4%, p = 0.065) and MUFA (namely, C20:1 and C22:1) contents, higher C22 n-3 PUFA contents, and higher C20:2n-6 (+85.1%, p = 0.059) and C22 n-6 PUFA contents. These analyses of foetal plasma fatty acids were confirmed by PCA since exposed and control F2 foetuses were discriminated by their fatty acid specific signatures (i.e., profiles) on the corresponding PCA, representing 61.6% of the inertia of the data in the main plan (Supplementary Fig. S6).

**Table 5:** Fatty acid profile in plasma in F2 of control (CC) and exposed (EC) fetuses at 28dpc (% of total fatty acids).

Variable	Number of fetus		Median [Q1; Q3]		Fully adjusted linear model		
	CC	EC	CC	EC	β value	CI	p-value
C15:1n-9	12	12	0.16 [0.12; 0.22]	0.28 [0.21; 0.29]	0.10	[0.04; 0.16]	0.019*
C16:0 (PA)	12	12	39.4 [37.7; 40.3]	35.1 [31.5; 37.7]	−4.36	[−7.18; −1.55]	0.022*
Total C16:1	12	12	6.3 [6.0; 6.9]	6.0 [5.3; 6.5]	−0.27	[−1.15; 0.60]	0.558
C18:0 (SA)	12	12	11.5 [11.2; 11.9]	10.7 [10.3; 11.3]	−0.44	[−2.20; 1.32]	0.637
C18:1n-7	12	12	2.0 [1.8; 2.1]	1.5 [1.3; 1.6]	−0.39	[−0.62; −0.17]	0.013*
Total C18:1	12	12	22.6 [21.1; 23.1]	18.5 [17.7; 22.5]	−1.64	[−4.83; 1.54]	0.346
C20:0	12	12	0.13 [0.11; 0.15]	0.80 [0.42; 1.15]	0.58	[0.18; 0.98]	0.029*
C20:1n-9	12	12	0.38 [0.32; 0.46]	1.33 [0.95; 2.03]	1.02	[0.59; 1.44]	0.003**
C20:1n-7	12	12	0.07 [0.05; 0.08]	0.475 [0.28; 0.70]	0.40	[0.23; 0.57]	0.003**
Total C20:1	12	12	0.46 [0.39; 0.53]	1.80 [1.23; 2.73]	1.42	[0.83; 2.00]	0.003**
C18:2 n-6 (LA)	12	12	11.1 [10.5; 12.1]	9.1 [8.6; 9.9]	−1.70	[−3.19; −0.21]	0.065
C18:3 n-3 (ALA)	12	12	0.57 [0.44; 0.67]	0.57 [0.49; 0.57]	−0.06	[−0.27; 0.15]	0.582
C18:4n-3	12	12	0.18 [0.15; 0.28]	0.32 [0.24; 0.51]	0.16	[0.07; 0.25]	0.013*
C20:2n-6	12	12	0.34 [0.29; 0.39]	0.62 [0.21; 0.93]	0.29	[0.04; 0.53]	0.059
C20:3n-6 (DGLA)	12	12	0.49 [0.45; 0.57]	0.36 [0.30; 0.42]	−0.13	[−0.20; −0.06]	0.012*
C20:4 n-6 (AA)	12	12	2.6 [2.5; 2.8]	1.6 [1.34; 1.83]	−1.03	[−1.37; −0.69]	0.001**
C20:4n-3	12	12	0.09 [0.06; 0.15]	0.19 [0.10; 0.26]	0.13	[0.04; 0.22]	0.032*
C20:5 n-3 (EPA)	12	12	1.4 [0.01; 1.7]	1.5 [0.05; 2.6]	0.02	[−0.97; 1.01]	0.973
C22:0	12	12	0.09 [0.07; 0.10]	1.48 [0.71; 2.00]	1.31	[0.79; 1.83]	0.003**
C22:1n-9	12	12	0.05 [0.04; 0.12]	0.39 [0.35; 0.68]	0.45	[0.24; 0.66]	0.006**
C22:4 n-6	12	12	0.12 [0.05; 0.16]	1.85 [0.72; 4.22]	2.12	[1.23; 3.01]	0.003**
C22:5 n-6	12	12	0.20 [0.16; 0.25]	1.03 [0.35; 2.64]	1.05	[0.18; 1.91]	0.054
C22:5 n-3 (DPA)	12	12	0.06 [0.04; 0.14]	2.02 [0.80; 3.99]	2.18	[1.39; 2.96]	0.002**
C22:6 n-3 (DHA)	12	12	0.18 [0.16; 0.21]	0.24 [0.15; 0.61]	0.15	[0.02; 0.27]	0.055
Total SFA	12	12	52.8 [51.3; 54.1]	50.2 [47.1; 52.5]	−2.11	[−4.89; 0.67]	0.184
Total MUFA	12	12	29.6 [28.1; 30.9]	27.8 [26.2; 31.2]	−0.22	[−3.42; 2.99]	0.898
Total n-6 PUFA	12	12	15.7 [15.0; 17.0]	16.3 [13.8; 17.7]	0.49	[−1.25; 2.23]	0.599
Total n-3 PUFA	12	12	1.8 [1.1; 2.8]	4.3 [1.8; 7.5]	2.53	[0.47; 4.58]	0.051
n-6/n-3 PUFA ratio	12	12	9.4 [5.6; 15.1]	3.8 [2.5; 7.6]	−7.68	[−10.84; −4.52]	0.005**
PUFA/SFA ratio	12	12	0.33 [0.31; 0.36]	0.41 [0.30; 0.56]	0.09	[0.004; 0.17]	0.084

*Data are expressed as Median[Q1;Q3], in % of total FA. AA: C20:4n-6, arachidonic acid; ALA: C18:3n-3, alpha-linolenic acid; DGLA: C20:3n-6, dihomo-gamma-linolenic acid; DHA: C22:6n-3, docosahexaenoic acid; DPA: C22:5n-3, docosapentaenoic acid; EPA: C20:5n-3, Eicosapentaenoic acid; LA: C18:2n-6, linoleic acid; MUFA: monounsaturated fatty acids; PA: C16:0, palmitic acid; PL: phospholipids; PUFA: polyunsaturated fatty acids; SA: C18:0, stearic acid; SFA: saturated fatty acids* “Statistics with *P < 0.05” and **P < 0.01.

### Placental transcriptome

Hierarchical clustering revealed limited differences in the F2 placental transcriptome between the EC and CC groups, as shown by the very short clustering scale (supplementary Fig. S7). This close similarity between F2 EC and CC placentas was confirmed by the small number of differentially expressed (DE) probes, transcripts and annotated genes (Supplementary Table S3). The microarray data have been registered in the Gene Expression Omnibus (GEO) under accession number GSE109831.

**Figure 1:**
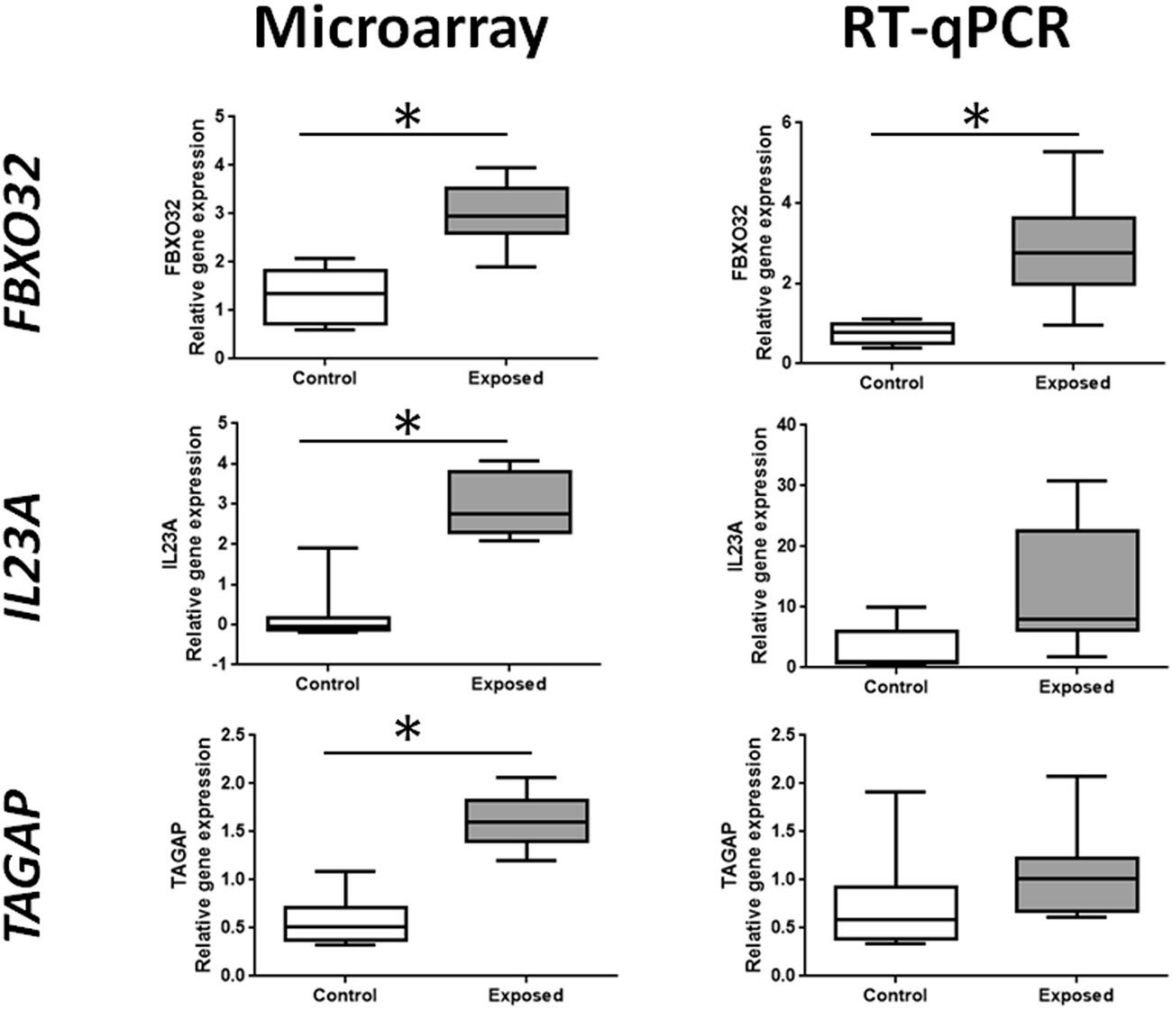
Expression of FBXO32, IL23A, and TAGAP in placentas of F2 of control (CC) and exposed (EC) conceptuses at 28dpc, whose mothers were or not *in utero* exposed to diesel engine exhausts, respectively. Expressions were analyzed by microarray (left column) and RT-qPCR (right column). Microarray data were first log2-transformed and then normalized by an intra-array median subtraction. Relative expression of genes obtained by RT-qPCR was normalized by the expression of two housekeeping genes, RPL18 and EIF4E2. Box-plots are representative of the median value and quartiles. Significant differences between conditions were figured out by * (for P value < 0.05).

Differential gene expression (adjusted p-value < 0.05) between EC and CC placentas was observed for F-box protein 32 (*FBXO32*, +119%, 4 significant probes on a total of 5 probes in microarray), T-cell activation RhoGTPase activating protein (*TAGAP*, +214%, 2 significant probes on a total of 2 probes) and Interleukin 23 α subunit p19 (*IL23A*, +275%, 1 significant probe on a total of 1 probe). After RT-qPCR, statistical significance was only found for one of the three transcripts highlighted by the microarray, FBXO32, confirming this result. For the other two transcripts, TAGAP and IL23A, only a trend going in the same direction was observed. (Fig. 1).

#### Gene set enrichment analyses (GSEA) in EC placentas

**Figure 2:**
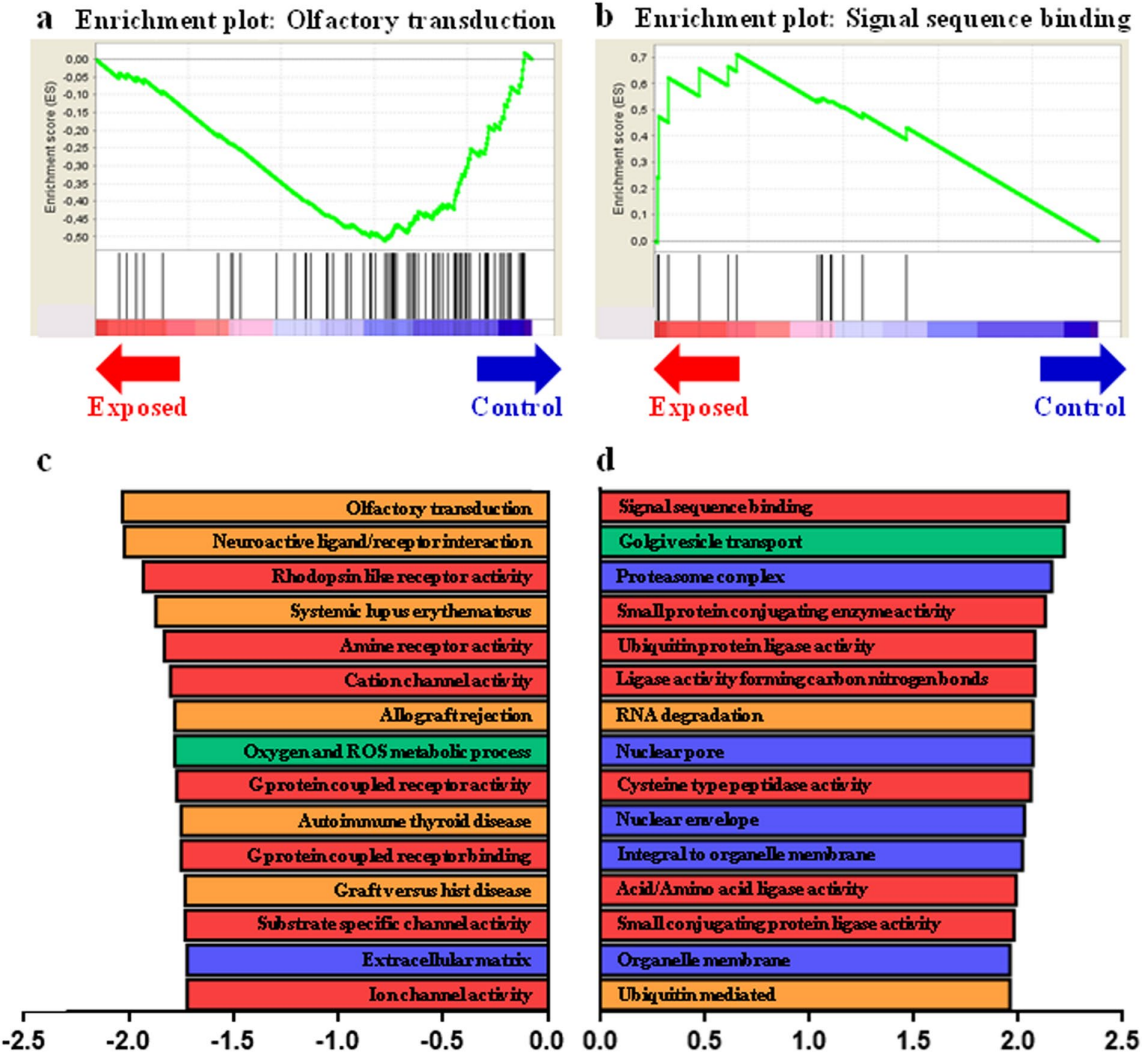
Gene Sets Enrichment Analysis in F2 of control and exposed placental labyrinthine area at 28dpc. GSEA-enrichment plot of the most negatively enriched gene set: Olfactory transduction. (**b**) GSEA-enrichment plot of the most positively enriched gene set: Signal sequence binding. (**c**) Bar plot depicting the normalized enrichment score of the top 15 most negatively enriched gene sets, constructed using several categories of 2 databases: C2CP:KEGG (Orange), C5:BP (Green), C5:CC (Blue) and C5:MF (Red). (**d**) Bar plot depicting the normalized enrichment score of the top 15 most positively enriched gene sets, which contains several databases C2CP:KEGG (Orange), C5:BP (Green), C5:CC (Blue) and C5:MF (Red).

Because the usual strategy used to analyse transcriptomics is based on a single-gene expression level that becomes inefficient with subtle variations in the same pathway^24^, we further analysed placental transcriptomic data using a GSEA approach as described above. Using GSEA, a total of 58 significantly enriched gene sets were observed after FDR multiple test adjustment (q-value < 0.25) for the EC group, including 10 terms for the KEGG (the Kyoto Encyclopedia of Genes and Genomes) database, 14 terms for the biological process (BP) category, 19 terms for the cellular component (CC) category and 15 terms for the molecular function (MF) category of the GO (Gene Ontology) database (Supplementary Tables S4–S7). In the top fifteen terms for all databases, using an enrichment score ranking, most terms defining the placenta-exposed group were related to intracellular organization (nuclear pore and envelope, “integral to organelle membrane”, “organelle membrane” and “Golgi vesicle transport”) and protein metabolism (“signal sequence binding”, “ubiquitin protein ligase activity”, “ubiquitin mediated proteolysis” and “proteasome complex”) (Fig. 2).

#### Gene set enrichment in CC placentas

A total of 69 significantly enriched gene sets were observed after FDR multiple test adjustment in the control group (i.e., comparatively downregulated pathways in EC placentas), including 16 terms in the KEGG database, 18 terms in the BP, 11 terms in the CC and 24 terms in the MF categories of the GO database (Supplementary Tables S4–S7). In the top fifteen gene sets enriched, most terms were involved in olfactory transduction and neuroactive ligand or in membrane and intracellular pathways (G protein coupled receptor activity, G protein coupled receptor binding, amine receptor activity, cation channel activity, substrate specific channel activity and ion channel activity) (Fig. 2).

## Discussion

This study was designed to explore maternal metabolism and placental function through several approaches to explain the previously observed foetal dyslipidaemia without biometric changes in F2 foetuses whose grandmothers were exposed to DE during gestation^7^. The two 1-hour daily exposures in F0 were used to mimic our home-work journeys morning and evening (corresponding to real life for outdoor air pollution exposure) particularly during the period of peak pollution in urbanized cities. The concentration of 1 mg/m^3^ for 2 hours translates to 85 µg/m^3^ when averaged over 24 hours, which is closer to the PM10 concentration that increases from 50 µg/m^3^ (European daily standard) to 100 µg/m^3^ and even higher during typical smog episodes in urbanized cities worldwide. The effects of *in utero* exposure to DE on the phenotype of exposed F1 rabbits were expected at adulthood according to the literature^25^. No significant metabolic modifications were observed in F1 females before pregnancy, but a trend towards hypertriglyceridemia only was expected (unpublished data); this finding was confirmed at 28 dpc since these pregnant F1 animals did not exhibit any difference in blood biochemistry or plasma fatty acid concentrations.

Gestation affects maternal lipid metabolism since maternal plasma free fatty acid (FFA) levels are commonly elevated in late pregnancy, participating in the onset of insulin resistance observed in pregnant women^26,27^, rodents^28^ and rabbits^29,30^. Thus pregnancy could be considered a metabolic challenge that could reveal latently impaired metabolism in rabbit females *in utero* exposed to DE. Since *in utero* exposure to polycyclic aromatic hydrocarbons (PAH), as potential actors in DE, causes hepatic steatosis in the female offspring of mice^31,32^, the liver was analysed in F1 females and hepatic steatosis was observed in exposed animals by histology, but could not be quantified firmly by this approach. Nevertheless, gas chromatography quantitative analyses confirmed that hepatic intracellular fatty acid concentrations were higher in exposed F1 females than in controls. Furthermore, the study of maternal plasma fatty acid profiles indicated that although both groups were given the same diet, plasma samples from exposed F1 females were enriched in C16:0 and mainly depleted in C16:1 and ALA. The increase in C16:0, parallel to decreased C14:0, could indicate higher *de novo* lipogenesis, which results in an increase of the DNL index (trend with p = 0.07) in plasma of F1 exposed dams^22,23^, probably using ALA as substrate^33^. The histological evaluation of placental vascularization and the trophoblast volume did not show any difference between EC and CC F2 placentas. The fatty acid concentration of placental membranes (phospholipid fraction) was not different between groups, thus confirming the stereological data. The placental intracellular lipid contents (neutral lipids), however, were greatly increased in EC compared to CC placentas, as also reported in placentas from pregnant rabbits fed a high fat diet^34,35^ but no effect of foetal sex could be found in any of the fatty acid analyses. Because EC placentas were enriched in fats, fatty acid profiles of F1 maternal and F2 foetal plasma samples were analysed. As expected, the fatty acid concentrations of F2 EC plasma were increased compared to those of CC, confirming previous plasma biochemical analyses showing higher plasma triglycerides in these foetuses^7^. As the placenta is a programming agent of foetal growth that is sensitive to the maternal environment, placental fatty acid profiles were investigated. In the phospholipid fraction of EC placentas, a weak decrease of non-biologically active intermediates of the n-6 polyunsaturated fatty acid (PUFA, namely, C20:2n-6 and C22:4n-6) was observed, as well as a slight decrease in saturated fatty acid (SFA) while monounsaturated fatty acid (MUFA, namely, C16:1) was increased, leading to a small increase in the PUFA/SFA ratio. These data suggest that membrane fluidity was increased^36^, possibly leading to increased fatty acid transfers across the placenta^37–39^.

Essential fatty acids to be transferred from dam to foetus, namely, arachidonic (AA, C20:4n-6), eicosapentaenoic (EPA, 20:5n-3) and docosahexaenoic acids (DHA, 22:6n-3), accumulate preferentially in placental phospholipids that act as a regulator of fatty acid uptake in trophoblastic cells prior to being transferred to the foetus^40^. Most potent eicosanoids are derived from AA, but additional biologically significant eicosanoids are also derived from dihomo-gamma-linolenic acid (DGLA), which is produced in the reaction pathway leading to AA from LA, with distinctly different biological actions (less inflammation or even anti-inflammation, vasodilation, and the inhibition of platelet aggregation^41^). In EC placentas, where fatty acid concentrations were nearly doubled compared to controls, the proportion of n-6 (C22:4n-6) and n-3 long chain PUFA (DPA and DHA) was increased in neutral lipids (lipid storage), while the percentage of AA was halved, remaining, however, sufficient to cover foetal needs.

In foetuses, plasma long chain PUFA concentrations depend on placental transfers^27,42,43^. Here, the AA content and n-6/n-3 PUFA ratio were decreased in the plasma of F2 EC fetuses. A high n-6/n-3 ratio usually reflects low-grade inflammation^44^. The increased proportion of n-3 PUFA in foetal plasma could explain the observed lower total and non-HDL cholesterol concentrations in the F2 EC foetal plasma, as observed in mice in a context of atherosclerosis and dietary n-3 PUFA intake^45^. Moreover, since plasma fatty acid concentrations were higher in EC foetuses compared to those of CC, this further increased the F2 EC foetal reserves of n-3 PUFA, among which biologically active PUFA (namely, EPA, DHA), were favoured by selective placental fatty acid transfer, whereas plasma AA was decreased, which in return limited pro-inflammatory processes. Since anti-inflammatory eicosanoids are derived from n-3 PUFA^43^, these changes in plasma F2 fatty acid profiles could be a positive adaptive response to counteract the effects of perturbed fatty acid metabolism in dams (as discussed above).

The placental transcriptomics analysis showed that only three genes were differentially expressed, namely, *interleukin 23 subunit alpha* (*IL-23A), T-cell activation Rho GTPase-activating protein (TAGAP) and F-Box Protein 32 gene (FBXO32*), among which only the last gene was confirmed after RT-qPCR.

The *IL23A* gene is expressed in placental trophoblastic cells and its over-expression was reported in the context of unexplained recurrent spontaneous abortion during early pregnancy^46^ or in miscarriage^47^. IL-23, which is produced, among others, by macrophages and dendritic cells, is an important component of the inflammatory response^48^, by promoting the upregulation of the matrix metalloproteases MMP-9, increasing angiogenesis and being involved in the Th17 cell differentiation^48^, and activating pro-inflammatory IL-17-producing cells in both mice and humans^49^. Urban Particulate Matters were shown to promote an inflammatory milieu in part by inducing an IL-23-driven pro-inflammatory Th17 response in co-cultured cells *in vitro*^50^.

TAGAP has been described to exert a regulatory role in T cell activation in Crohn’s disease^51^.

*FBXO32* encodes atrogin-1 (mostly expressed in muscular tissues), a member of the F-box protein family, one of the four subunits of the ubiquitin protein ligase complex, whose function is central for the selection of proteins targeted for degradation by the ubiquitin proteasome pathway^52^. In pregnant mice, food deprivation reduced FBXO32 gene expression in the placenta^52^, whereas its expression was increased in high-fat diet-fed turtles^53^ or mice^54^. These genes were upregulated in EC compared to CC placentas, but the physiological meaning of their increase remains to be determined.

A GSEA analysis was performed to identify biological pathways enriched in genes associated with grandmother exposure. This approach showed that 58 gene sets were enriched in EC placentas and 69 in CC placentas. In EC placentas, “proteasome complex”, “ubiquitin protein ligase activity” and “ubiquitin mediated proteolysis” gene sets were enriched. The proteasome is a protein-destroying apparatus involved in many essential cellular functions, such as the regulation of the cell cycle, cell differentiation, signal transduction pathways and apoptosis. The proteasome is involved in degrading a variety of cellular proteins in a rapid and timely way and most substrate proteins are modified by ubiquitin before their degradation by the proteasome^55^. The slight over-expression of genes encoding the protein degradation machinery in EC placentas, together with the over-expression of the *FBXO32 gene* involved in the formation of the proteasome, suggests an increase in protein degradation following either an increase in protein production or an increase in malformed and misfolded proteins. The over-expression of the proteasome complex component has been associated with premature birth^56^ or preeclampsia^57^ in humans. In intrauterine growth retardation, the ubiquitination of the amino acid transporter SNAT2 was reported to be increased^58^, leading to decreased SNAT2 protein expression and decreased placental amino acid transport. Here, growth retardation, however, was not observed. The « RNA degradation » gene set was also enriched in EC placentas and comprised genes encoding proteins involved in the carbon catabolite-repression 4-Not complex (CCR4-NOT) that regulates mRNA synthesis and degradation as well as exosome trafficking^59^. The over-expression of genes involved in the RNA degradation machinery was associated with the enrichment of “nuclear pore” and “nuclear envelope” gene sets (involved in RNA transport through the nuclear membrane) and with the enrichment of “organelle membrane”, “integral to organelle membrane” and “Golgi vesicle transport” gene sets (genes participating in protein transport between the cellular organelles). Altogether, these observations point to an increase in cellular protein and RNA trafficking. The “cation/ion channel activity” gene sets were downregulated in EC placentas. Finally, “systemic lupus erythematosus”, “allograft rejection”, “graft versus host disease” and “autoimmune thyroid disease” gene sets were downregulated in EC placentas. These gene sets include genes encoding for proteins involved in pro-inflammatory response and immune reactions with lymphocyte activation and TNF, IL10, and IL1A secretion. Although IL23A and TAGAP do not belong to these gene sets, these results are consistent with the general picture derived from FA analyses, suggesting that some protection against inflammatory processes is induced in F2 placentas from the exposed group.

In summary, *in utero*-exposed female rabbits showed an impaired metabolic phenotype in adulthood, and once pregnant, these animals exhibited liver steatosis, likely underlining a deregulation of lipid metabolism. Moreover foetuses from these females were dyslipidaemic and compared to controls, their placental lipid contents were higher, suggesting that these placentas cannot succeed in protecting the foetuses from maternal dyslipidaemia. However, the fatty acid concentrations and profiles of the placenta suggest adaptive protective mechanisms against inflammation through the placental storage of non-essential fatty acids. This mechanism was illustrated by MUFA storage and the favoured transplacental transfer of n-3 PUFA, which are known for their lipid-lowering effects and anti-inflammatory roles. Moreover, these placentas appeared programmed to counteract any inflammatory exposure as shown by a decrease in the pro-inflammatory arachidonic acid (AA) content and its consequent decrease in the foetal blood compartment as well. These results are consistent with the molecular changes observed in F2 placentas using GSEA analysis of transcriptomic placental data, showing a downregulation of gene sets involved in inflammation, coherent with the upregulation of gene sets involved in protein metabolism (proteasome) and intracellular signalling. Contrary to what has been shown elsewhere, for example, in the case of maternal lipid over-nutrition in rabbits^35^, suggesting that male and female placental responses differ, this was not the case here. Foetal sex has been taken into consideration in all of the statistical analyses, as an explicative factor, but no sex-related difference reached statistical significance in any of our datasets. This finding may be explained by the fact that compared with F1 controls, *in utero* exposure did not lead to observable differences in body weight or blood biochemistry; moreover, in adulthood, the F1 females were all fed with the same balanced and optimal gestation-specific diet. Thus, conditions might not have been met metabolically to induce or highlight different gender-specific responses or that the effects of pollution may have been much stronger than the sex-specific responses that would then have been either smoothed or erased. However, it cannot be excluded that the sample size was too small to highlight sex-specific effects. The authors acknowledge that this experimental study, which was continued until the F2 generation, has limitations due to small sample size for the control group, especially due to gestation failure. Thus although the authors have confidence in their results, these results must be considered with circumspection and therefore remain preliminary.

## Conclusion

In conclusion, the indirect exposure of F1 females *in utero* to DE might affect placental function and foetal metabolism in their F2 offspring at ended gestation. Different technical approaches, using membrane biochemistry and molecular biology, have demonstrated potentially favourable placental counter-regulation around inflammation to preserve F2 foetuses. Altogether, these preliminary results demonstrate that maternal exposure to air pollution due to diesel engine exhaust should be considered as a programming factor within the context of the Developmental Origins of Health and Diseases (DOHaD), with a high risk of intergenerational transmission. The mechanisms involved in this transmission and the long-term health effects of F2 after birth remain to be determined.

## Materials and Methods

### Animals

All experimental protocols were approved by the local ethical committee COMETHEA (*Comité d’Ethique en Expérimentation Animale du Centre INRA de Jouy en Josas et AgroParisTech*), referenced as N°C2EA-45 in the French National registry CNREEA (*Comité National de Reflexion Ethique sur l’Expérimentation Animale*), under N°12/102. The methods were carried out in accordance with the relevant guidelines and regulations. As described previously, F0 pregnant New Zealand white female rabbits (INRA1077 line) were exposed by nose-only inhalation to diluted DE (exposed group) or clean air (control group) at a concentration of 1 mg/m^3^ with a mean particle diameter of 69 nm for 2 h/day and 5 days/week, from the 3^rd^ to the 27^th^ day post-conception (dpc)^7^. Briefly, DE exposure was performed with the Mobile Ambient Particle Concentrator Exposure Laboratory^60^ connected to a 25KVA Loxam engine with a 500 nm particle filter. The measured components of the exposure mixture used in the present experiment were published previously^7^. Sixteen F0 females (N = 7 Controls, N = 9 Exposed dams) gave birth to F1 offspring, which were raised under control conditions. Altogether, 72 F1 offspring survived until adulthood, including 18 control females, 40 exposed females, 11 control males and 16 exposed males. All animals were weaned at 5 weeks of age. After becoming sexually mature adults at 6.5 months of age, a limited number of F1 nulliparous females (11 F1 control and 11 F1 exposed females) were dedicated to the production of the F2 generation. These females were mated with unexposed males. In the controls, only 5 out 11 rabbits were diagnosed as pregnant, among which one rabbit died at mid-pregnancy and another aborted; in the exposed group 10 out 11 rabbits were pregnant, including 1 abortion at mid-pregnancy. All females that were still pregnant (N = 3 controls and N = 9 exposed) were euthanized at 28 dpc to collect second generation (F2) foeto-placental units (see experimental protocol on Supplementary Figure S8). Maternal and foetal plasma were also collected and frozen at −80 °C until further assay. As a reminder, in this study hereafter, CC refers to F2 foetuses collected from F1 control dams (N = 21) and EC refers to F2 foetuses collected from *in utero*-exposed F1 dams (N = 87). Foetuses were weighed; foetal crown-rump length, biparietal diameter and head length (using a digital caliper) and abdominal diameter were measured prior to dissection. Blood samples, sampled prior to sexing, were collected from all foetuses. During dissection, each foetus was sexed by visual observation of the internal genital organs and organs were subsequently collected and weighed. Two males and two females were retained per litter for the analyses; we have sexed the foetuses beginning from the left horn on the ovarian side towards the right horn, and we collected the samples of the first two males and two females among the dissected specimens. The labyrinthine area of the placenta (materno-foetal exchange zone, of foetal origin) was dissected from the placental junctional area and the decidua, and split for either fixing or snap freezing in liquid nitrogen for further analyses. Analyses were all made on samples from the same male-female couples per litter (2 couples/ CC litter and 1 couple/ EC litter), as follows: maternal plasma and hepatic samples (3 C and 9E), placental and foetal plasma samples (12 CC and 12 EC, randomly chosen among the collected ones, half females and half males) for biochemistry and fatty acid analyses. The histological and stereological evaluation of the labyrinthine area of the placenta were limited to 6 placental samples per group, randomly chosen from the same sampling assortment as those used just above. Similarly, 8 different placental samples were randomly chosen among the selected samples for transcriptomic analyses (eight replicates per condition).

### Biochemistry

Classical biochemistry (triglycerides, cholesterol, glycaemia, urea, creatinine, ASAT, ALAT) was performed on maternal (n = 3 C and 9 E) and fetal EDTA-plasma (n = 12 CC and 12 EC) using a classical automated biochemistry analyser (DXc800 Beckman Coulter equipment). Plasma insulin was assayed with chemiluminescent immunoassay using paramagnetic particles on an automaton analyser (DXi Beckman Coulter equipment).

### Fatty acid analysis

A total of 100 µL of maternal plasma (n = 3 C and 9 E), 100 µL of foetal plasma (n = 12 CC and 12 EC) and 300 mg of the labyrinthine area (n = 12 CC and 12 EC) from each selected conceptus and of maternal livers were used for lipid analysis. After classical lipid extraction of plasma^61^, fatty acids were transmethylated with BF3-methanol and the fatty acid methyl esters (FAME) were analysed by Gas Chromatography-FID (GC 3900 Varian, France) on an Econo-Cap EC-WAX capillary column^35^. After lipid extraction of the placental tissue, the phospholipids (tissue membranes) were separated from neutral lipids (intracellular lipid store) using silica acid cartridges (Supelco, Bellefonte, PA) according to Juaneda *et al*.^62^ prior to transmethylation of the fatty acids from these two fractions and gas chromatography analysis^63^. The identification of fatty acids was made in reference to known fatty acid profiles obtained from injection of standard FAME mix (Supelco 37 components FAME mix, ref 47885-U, Sigma). The fatty acid profile was established for each sample and expressed as a % of the total fatty acids. Heptadecanoic acid (C17:0), introduced prior to the plasma lipid extraction (50 µg) or prior to the fatty acid transmethylation of placental phospholipid and neutral lipid fractions (200 µg), was used as an internal standard to measure the total fatty acid concentration of plasma or for relative fatty acid quantification of each placental lipid fraction after chromatogram analysis. The fatty acids were identified using chromatographic analyses as described in the Supplementary Note. The DNL index was determined in maternal plasma as de novo lipogenesis (C16:0/C18:2n-6 ratio)^22,23^. Only a few relevant fatty acids are outlined in the result tables, but all the fatty acids are included in reported totals. Moreover, Principal Component Analyses (PCA) were performed using the full fatty acid profiles (*cf*. Statistics).

### Liver histology

Maternal liver fragments from all F1 dams were fixed in 4% formaldehyde for at least 10 days prior to dehydration in ethanol solutions, clearing in butanol, embedding in paraffin and cutting into 5 µm thick sections (Leica microtome, Germany). Four different colourations were performed on liver sections: a routine hematoxyline eosine Safran (HES) colouration to reveal the different cellular components, a Prussian blue coloration (Perl’s colouration) to highlight possible ferric deposits and the activation of Kupffer cells, a Masson’s trichrome colouration (Microm Microtech France) to visualize collagen fibres and a periodic acid Schiff (PAS) staining to highlight the presence of glycogen. All coloured sections were analysed under light microscopy and scanned using the Nanozoomer Digital Pathology System (NDP Scan U10074-01, Hamamatsu, Japan).

### Immunohistochemistry

**S**amples of the labyrinthine area (N = 6 CC and N = 6 EC) were fixed with 4% formalin. After dehydration in ethanol solutions and washing in xylene, and the samples were embedded in paraffin and cut into 7 µm thick sections (Leica microtome, Germany). Immunodetection of vimentin was performed to label foetal capillaries as described previously^35^.

### Stereological analyses

The cellular composition of the labyrinthine area was explored by stereology. After vimentin immunodetection, all placental sections were scanned using the NanoZoomer Digital Pathology System (NDP Scan U100074-01) as previously described^64^. The volume fraction and surface density of trophoblasts, foetal vessels and maternal blood space, i.e., the components of the labyrinthine area, were quantified using the One Stop Stereology method available on Mercator® software, as described previously^65^.

### Transcriptomic analyses

#### RNA isolation

Total RNA was extracted from frozen pieces of labyrinthine area in liquid nitrogen, randomly collected from each control (n = 8 CC), exposed (n = 8 EC) litter, half males and half females. The samples were ground in a solution D + (namely: thiocyanate guanidine 4 M, sodium citrate 25 mM pH 7, N-lauryl-sarcosine 0.5%, β-mercaptoethanol 0.1 M). The RNA was extracted twice with 3 M sodium acetate pH 4–5, aquaphenol and chloroform/isoamylic ethanol (24:1), then precipitated in isopropanol at −80 °C overnight. A purification procedure using DNAse I (Qiagen) treatment at 25 °C for 15 min was performed prior to elution. The RNA pellet was washed with ethanol, dried and solubilized in RNase-free water (10 µl of H_2_O for 100 mg of tissue). The RNA was purified with the RNeasy Mini Kit (Qiagen) and quantified by Nanodrop (Thermo Scientific, Madrid, Spain), and the quality was verified on the Bioanalyzer 2100 (Agilent Technologies). Total extracted RNA was stored at −80 °C for further RNA labelling.

#### RNA amplification and labelling and microarray processing

The eight samples from each group were used as biological replicates for the microarray analysis. RNA from each placental tissue (200 ng) was amplified and labelled with cyanine 3 (Cy3) dye using the One-colour Microarray-based Gene Expression Analysis Low Input Quick Amp Labelling Kit (Agilent Technologies). Excess dye was removed with the RNeasy Mini Kit (Qiagen) and dye incorporation and concentration were determined using the microarray setting on the Nanodrop 2000 and the Bioanalyzer 2100. Hybridization was performed at the CRB GADIE (INRA Jouy-en-Josas, France, http://crb-gadie.inra.fr/). Equal amounts of Cy3-labelled samples (600 ng, Specific Activity > 6.0 pmol Cy3/μg of cRNA) were mixed with blocking agent and fragmentation buffer, and then 24 μl of the mixture was hybridized into the Rabbit Custom Gene Expression Microarrays (GEO accession GPL18913, Agilent-042421 Rabbit BDR version 2, Agilent Technologies) for transcriptomic analysis (12775 genes)^66^. After 17 h at 65  °C, the hybridized slides were washed twice for 1 min at room temperature in GE Wash Buffer 1 and 1 min at 37 °C with GE Wash Buffer 2 (Agilent Technologies) and air-dried and scanned immediately.

#### Microarray analysis

After hybridization, the scanned images were analysed using Feature Extraction software (v10.7.3.1; Agilent Technologies). The data were normalized using intra-array median subtraction and log2 transformation. The raw data intensity files were read into R (www.r-project.org). The identification of differentially expressed genes from EC and CC F2 placentas was achieved using the Limma package^67^. P values obtained by this analysis were adjusted for multiple testing using the Benjamini-Hochberg procedure. Probes with an adjusted P value < 0.05 were considered significant.

#### Gene Set Enrichment Analysis (GSEA)

The standard strategy to analyse transcriptomic data is based on a single-gene level that is very efficient when perturbations exert strong impacts on individual genes. This classical approach, however, becomes inefficient with subtle conditions because only a minor fraction of the information contained in whole-genome transcriptomic dataset is used, due to arbitrary thresholds used^24^. Gene set enrichment analysis (GSEA) was established for use in metabolic studies in the 2000s^68^. All detected unfiltered transcripts are mapped to their intended pathways (or other “sets of interest”). The subsequent analysis within specific pathways enables the study of the regulation of specific pathways that include moderately regulated (and non-regulated) genes usually lost due to experimental background noise. Here, the GSEA approach^68^ was used on transcriptomic data to systematically identify biological pathways enriched in genes associated with F1 foetal exposure and performed using the following parameters: 1,000 gene set permutations, weighed enrichment statistics, gene set size between 15 and 500 and signal-to-noise metrics. Regulated pathways were considered statistically significant when the false discovery rate (FDR) q-value was <0.25. The GSEA-derived normalized enrichment score was used for the visualization of pathway regulation, using the KEGG (The Kyoto Encyclopedia of Genes and Genomes) and GO (Gene Ontology) databases. KEGG is a database resource for understanding high-level functions and utilities of the biological system, from molecular-level information, especially large-scale molecular datasets generated by high-throughput experimental technologies. One of the main uses of GO is to perform enrichment analysis on gene sets. For example, given a set of genes that are up-regulated under certain conditions, an enrichment analysis will find which GO terms are over-represented (or under-represented) using annotations for that gene set. Thus C2:KEGG and several categories of the more detailed GO gene set database, i.e. C5:BP (Biological Processes), C5:CC (Cellular Component) and C5:MF (Molecular Function)^69^, were used to identify the top 15 pathways characterizing each group.

#### Reverse transcription and real-time PCR

For validation of the microarray experiments, total RNA was extracted from pieces of the labyrinthine area from the same control (n = 8 CC) and exposed (n = 8 EC) placentas. The RNA was isolated on silica-based columns, using the RNeasy Plus Mini Kit with the effective elimination of genomic DNA, following the manufacturer’s instructions (Qiagen, France). Total RNA was quantified using OD 260 nm on a NanoDrop 1000 spectrophotometer (ThermoScientific, Baltimore, MA, US) and quality was assessed with the Fragment Analyzer (AATI, US) to determine the RNA quality number. RNA (750 ng) was reverse-transcribed using the iScript Reverse Transcription Supermix (Bio-Rad, Hercules, CA, US). The expression of conventionally used housekeeping genes, including RPL18 and EIF4E2, was shown to be stable between assays. Quantitative real-time PCR (qRT-PCR) was performed with the Fast SYBR Green Master Mix and the Applied Biosystems 7900HT Fast Real-Time PCR System (Applied Biosystems, Foster city, California US). The experimental protocol consisted of an initial polymerase activation at 95 °C, for 20 s followed by an amplification program for 40 cycles maintaining the annealing and extension primer temperature at 62 °C for 20 s. A melting-curve analysis was then performed to verify the amplification of a single product. All primers were designed using NCBI Primer-BLAST and selected to generate amplicons with a length of 100–200 bp (Table 6). The primers were synthesized by Eurogentec. Standard curves were generated to calculate the efficiency of each set. Only primer sets with an efficiency of 1.85 to 2.1 were used for qPCR. The relative mRNA levels for each assay were computed from the Ct values obtained for the target gene. The data were pooled and analysed using RQ manager v1.2 and dataAssist v1.0 (Applied Biosystems). DataAssist™ software is a simple, yet powerful data analysis tool for sample comparison when using the comparative cycle threshold (CT) method^70^ for calculating the relative quantitation of gene expression. This software contains a filtering procedure for outlier removal, various normalization methods based on single or multiple genes, and provides relative quantification analysis of gene expression through a combination of statistical analysis and interactive visualization.

**Table 6:** Primers (DNA oligos) used for FBXO32, IL23A and TAGAP mRNA quantification by real-time RT qPCR, in *Oryctolagus cuniculus species*.

Genes	Accession number	Sequences of forward (FW) and reverse (RV) primers	
FBXO32	XM_002710762	L-FBXO32-FW	CCT-CCA-GCT-CTG-CAA-GCA-TT
		L-FBXO32-RV	AAA-GTC-CTG-GGG-TGA-AAG-TGA
IL23A	XM_002711079	L-IL23A-FW	TGG-GAC-CCA-TGG-AGC-TAC-TA
		L-IL23A-RV	TGC-AGG-CAG-AAC-TGA-GTG-TT
TAGAP	XM_008263738	L-TAPAG-FW	GGG-AAG-GTT-AAG-ACG-CTG-GT
		L-TAPAG–RV	GGT-GGA-CGT-GTC-TGA-ACT-GT
EIF4E2	ENSOCUG00000004534	L-EIF4E2-FW	TGG-CAA-GTG-GAT-TAT-TCG-GC
		L-EIF4E2-RV	CAG-AGA-CCA-CAG-CCC-CAC-AG
RPL18	DQ403030	L-RPL18-FW	CAA-CTC-CAC-GTT-CAA-CCA-GGT
		L-RPL18-RV	GGT-CTT-GTT-CTC-CCG-CCC

*Endogenous transcripts, EIF4E2 and RPL18, were used as reference transcripts*.

### Statistical analyses

All data (except placental transcriptomic data) are expressed as median[Q1;Q3], with the first (Q1) and third quartiles (Q3) corresponding to 25 and 75% of the scores, respectively. For all F1 maternal data, a linear model was used, with random effect of dams adjusted for treatment, birth date (indexed in 3 categories), integrating neonatal and peri-weaning data of these F1 females to account for their early nutritional status (litter size at birth and at weaning) using the linear mixed effects model (nlme package, R, Pinheiro, Bates, DebRoy, Sarkar and the R Development Core Team 2013. nlme. R package version 3.1–111; http://www.r-project.org/). The β-value represents sample variability after adjustment. A negative β-value represents a reduction in the exposed group and a positive β-value represents an increase in the exposed group compared to controls. A similar linear model was used for F2 foetal data with a random effect of dams adjusted for treatment, litter size, foetal position in the horn (indexed in 3 categories - A, B or C – to indicate the position of the foetus in the horn, with A: at the top of the horn near the ovary, B: in the middle of the horn or C: at the bottom of the horn towards the vagina) and foetal sex as an explicative factor. For fatty acid profiles, linear mixed models identical to those used for maternal and foetal data were used to analyse each fatty acid for each profile. Principal Component Analyses (PCA) were performed using FactoMineR (R package^71^ dedicated to multivariate data analysis^69^) to analyse the biological relevance of the significant differences observed using the linear model.

### Ethical approval

All experiments were carried out in accordance with relevant guidelines and regulations. All experimental protocols were approved by the local ethical committee, referenced as N°45 in the French National register, under N°12/102.

## Supplementary information

Supplementary data

Supplementary Table S3

## Data Availability

The datasets supporting the conclusions of this article are included within the article and its Supplemental Files; microarray data were registered in Gene Expression Omnibus (GEO) under accession number GSE109831.
