# Supplementary material for: Effects of first-generation in utero exposure to diesel engine exhaust on second-generation placental function, fatty acid profiles and foetal metabolism in rabbits: preliminary results
Source: Sci Rep. 2019 Jul 4;9:9710. doi: 10.1038/s41598-019-46130-x (PMC6609606; doi:10.1038/s41598-019-46130-x)
Supplement: Supplementary file 1 — Supplementary data [file 41598_2019_46130_MOESM1_ESM.pdf]

# Effects of first-generation in utero exposure to diesel engine exhaust on second-generation placental function, fatty acid profiles and foetal metabolism in rabbits: preliminary results

Delphine Rousseau-Ralliard[1,2¶*], Sarah A. Valentino[1,2¶], Marie-Christine Aubrière[1,2], Michèle Dahirel[1,2], Marie-Sylvie Lallemand[1,2], Catherine Archilla[1], Luc Jouneau[1], Natalie Fournier[3,4], Christophe Richard[1,2], Josiane Aioun[1,2], Anaïs Vitorino Carvalho[1], Lecardonnel Jérôme[5], Rémy Slama[6], Véronique Duranthon[1], Flemming R. Cassee[7,8], Pascale Chavatte-Palmer[1,2¶], Anne Couturier-Tarrade[1,2¶*]

[1] UMR BDR, INRA, ENVA, Université Paris Saclay, Jouy en Josas, France

[2] PremUp Foundation, Paris, France

[3] Univ. Paris-Sud, EA 4041/4529 Lip (Sys)2, UFR de Pharmacie, Châtenay-Malabry, France

[4] Hôpital Européen Georges Pompidou (AP-HP), Laboratoire de Biochimie, UF Cardio-Vasculaire, Paris, France

[5] GABI CRB GADIE, INRA, Université Paris Saclay, Jouy en Josas, France

[6] Inserm, Univ. Grenoble Alpes, CNRS, IAB joint Research Center, Team of Environmental Epidemiology Applied to Reproduction and Respiratory Health, Grenoble, France

[7] Centre for Sustainability, Environment and Health, National Institute for Public Health and the Environment, Bilthoven, Netherlands

[8] Institute of Risk Assessment Sciences, Utrecht University, Utrecht, Netherlands.

[*] **Corresponding author.**

E-mail: delphine.rousseau@inra.fr and anne.couturier-tarrade@inra.fr

[¶] The co-first Author Sarah A. Valentino has provided equal contribution as the first author.

**Supplementary information online**

## Supplementary note: Fatty acid nomenclature

| symbol | Systematic name | Trivial name |
|---|---|---|
| C14:0 | Tetradecanoic acid | Myristic acid |
| C14:1 | cis-9-Tetradecenoic acid | Myristoleic acid |
| C15:0 | Pentadecanoic acid | Pentadecanoic acid |
| C15:1 | cis-10-Pentadecenoic acid | Pentadecenoic acid |
| C16:0 | Hexadecanoic acid | Palmitic acid |
| C16:1n-7 | cis-9-Hexadecenoic acid | Palmitoleic acid |
| C18:0 | Octadecanoic acid | Stearic acid |
| C18:1n-9 | cis-9-Octadecenoic acid | Oleic acid |
| C18:1n-7 | cis-11-Octadecenoic acid | Vaccenic acid |
| C18:2n-6 | trans,cis-10,12-Octadecadienoic acid | Linoleic acid (LA) |
| C18:3n-6 | cis,cis,cis-6,9,12-Octadecatrienoic acid | Gamma-linolenic acid (GLA) |
| C18:3n-3 | cis,cis,cis-9,12,15-Octadecatrienoic acid | Alpha-linolenic acid (ALA) |
| C20:0 | Eicosanoic acid | Arachidic acid |
| C20:1n-9 | cis-9-Icosenoic acid | Gadoleic acid |
| C20:2n-6 | cis-11,14-Eicosadienoic acid | Eicosadienoic acid |
| C20:3n-6 | cis-8,11,14-Eicosatrienoic acid | Dihomo-$\gamma$-linolenic acid (DGLA) |
| C20:4n-6 | cis,cis,cis,cis-5,8,11,14-Icosatetraenoic acid | Arachidonic acid (AA) |
| C20:5n-3 | cis,cis,cis,cis,cis-5,8,11,14,17-Icosapentaenoic acid | Timnodonic acid (EPA) |
| C22:0 | Docosanoic acid | Behenic acid |
| C22:1n-9 | cis-13-Docosenoic acid | Erucic acid |
| C22:4n-6 | cis,cis,cis,cis-7,10,13,16-Docosatetraenoic acid | Adrenic acid |
| C22:5n-6 | cis,cis,cis,cis,cis-4,7,10,13,16-Docosapentaenoic acid | Osbond acid |
| C22:5n-3 | cis,cis,cis,cis,cis-4,8,12,15,19-Docosapentaenoic acid | Cuplanodonic acid (DPA) |
| C22:6n-3 | cis,cis,cis,cis,cis,cis-4,7,10,13,16,19-Docosahexaenoic acid | Cervonic acid (DHA) |
| C24:0 | Tetracosanoic acid | Lignoceric acid |
| C24:1n-9 | cis-15-Tetracosenoic acid | Nervonic acid |

## Supplementary figures online

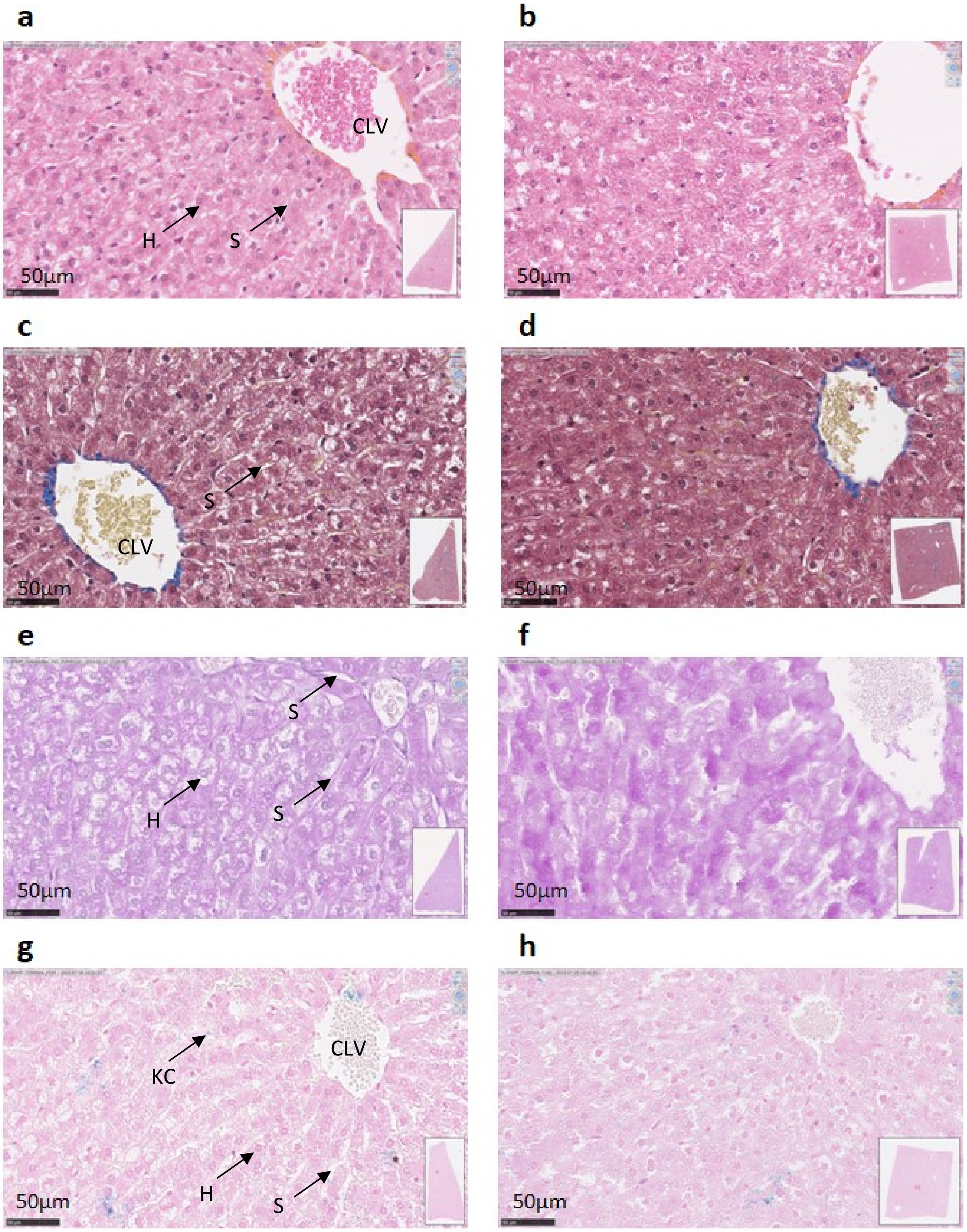


**Supplementary Figure S1 online: Histological analyses of F1 maternal liver.**

5µm thick sections of liver slices from *in utero* exposed F1 pregnant rabbits (n=6) on the left side or control F1 pregnant rabbits (n=3) on the right side. These liver sections were colored with **(a and b)** Hematoxyline Eosine Safran (HES) coloration to reveal the different cellular components, (**c and d**) a

Masson Trichrome coloration (Microm Microtech, France) to visualize collagen fibers, (**e and f**) a PAS (Periodic Acid Schiff) staining to highlight the presence of glycogen, and (**g and h**) a Prussian blue coloration (Perl's coloration) to highlight possible ferric deposits and activation of Kupffer cells (**KC**). All colored sections were analyzed under light microscopy and scanned using a Nanozoomer Digital Pathology System (NDP Scan U10074-01, Hamamatsu, Japan). At this x40 magnification, we can observe hepatic parenchyma and the arrangement of hepatocytes (**H**). They form flattened, anastomosing blades, the thickness of which is of a single cell, and between which the blood circulates slowly towards the centrilobular vein (**CLV**). The sinusoids (**S**) are bordered by a discontinuous layer of endothelial cells, which do not rest on any basement membrane and which are separated from the hepatocytes by a small space (Disse space); it drains into the lymphatic portals. The cytoplasm of hepatocytes is highly eosinophilic, due to the presence of numerous mitochondria, with very fine basophilic granulations linked to numerous free ribosomes and granular endoplasmic reticulum. The stained section with PAS shows the presence of glycogen grains, which, being polysaccharides, are PAS-positive (colored in magenta), in control sections mostly.

Liver sections from *in utero* exposed (E) F1 dams, once pregnant at 28 dpc, showed marked microvacuolar steatosis, with micro and macro lipid vacuoles inside hepatocyts and heterogeneous dilated sinusoids, without fibrosis (**a, c, e and g**);

Liver slice from control (C) F1 dams, once pregnant at 28 dpc, showed moderated microvacuolar steatosis (**b, d and h**) and mostly glycogen overload using PAS coloration (**f**).

---

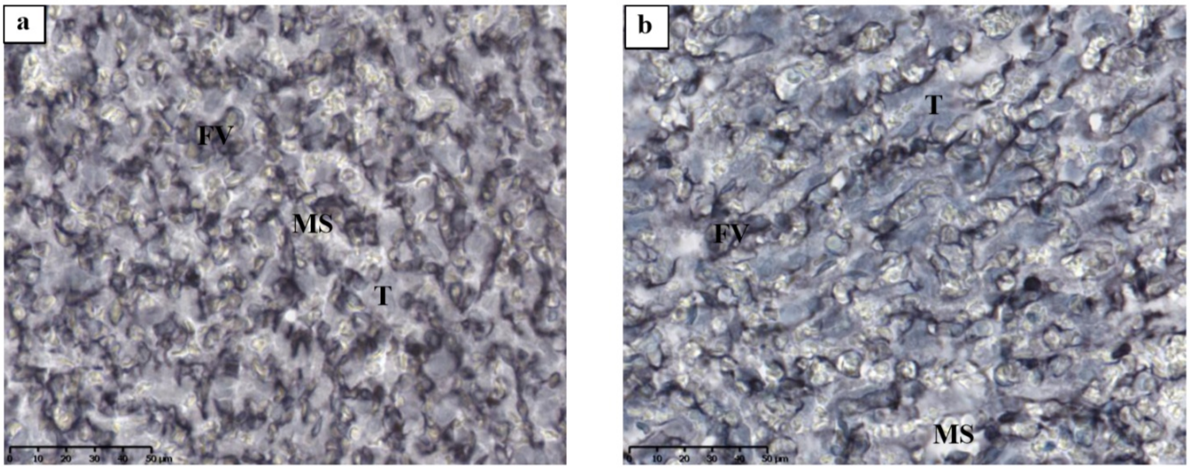


**Supplementary Figure S2 online: Stereological examination of F2 placenta at 28 dpc.**

At 28dpc, immunodetection of vimentin to label fetal capillaries was performed on labyrinthine area sections from the control group (a) and the exposed group (b). Black immunostaining represents fetal vessels (FV), blue cells are trophoblasts cells (T) and the white spaces with erythrocytes are maternal space (MS).

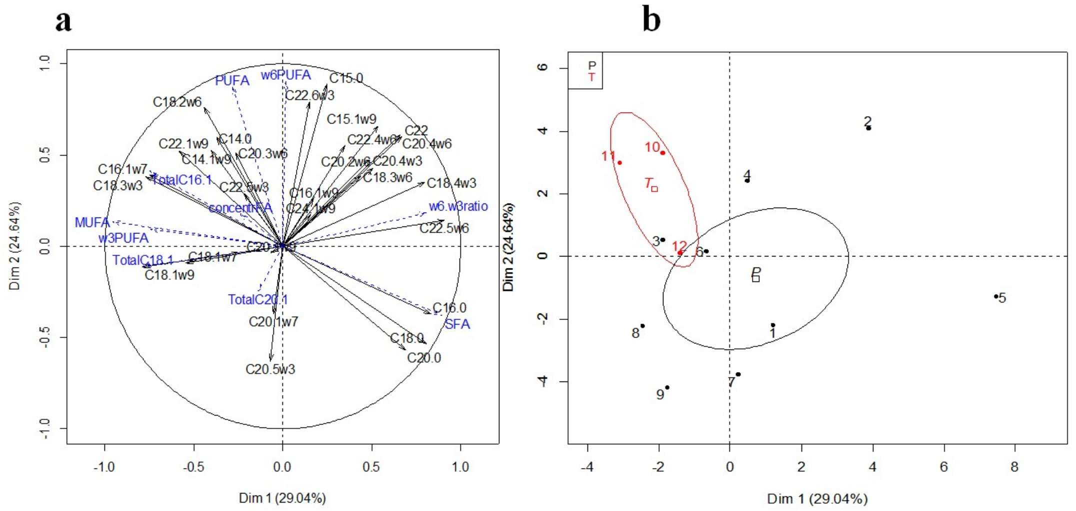


**Supplementary Figure S3 online: Principal Component Analyses (PCA) of the fatty acid profile in plasma of F1 pregnant female rabbits at 28dpc.**
The PCA, plotting individual factor scores, provides a representation in a space of reduced dimensions, thus providing data structure and highlighting groups of homogeneous individuals. For this, we have looked for sub-areas in which projection of the cloud deforms as little as possible the initial data summarized in Table 2: Fatty acid profiles of F1 maternal plasma (with FA expressed as % of total FA). The first principal axis is the one that maximizes the variance when data are projected onto a line and the second one is orthogonal to it, and still maximizes the remaining variance. The variables, here the fatty acids of the plasma profile, are plotted into a so-called Variables factor map (or "correlation circle" of the PCA, (**a**)), where the angle formed by any two variables represented as vectors, reflects their actual pairwise correlation. The Variables factor map allows identifying the variables that contribute "positively" vs. "negatively" to the PCA axes. The main plan, designed by the dimensions 1 and 2, represents 53.7% of the inertia of the data table, summarized in Table 2. The individual factor map (**b**) of this PCA shows that, as illustrated by the confidence ellipses drawn around the individuals, no specific FA signature characterized each group according to their *in utero* exposure, exposed (black) or control (red), since the dimension 1 does not correlate with the *in utero* treatment of the F1 female (v.test < 2). The main contribution of FA to dimension 1 concern a positive correlation (only the correlations >0.80 are reported here) with C22:5n-6 (0.91, p=4.4e-05), SFA (0.89, p=9.88e-05), C16:0 (0.83, p=8.38e-04), n-6/n-3 ratio (0.81, p=1.57e-03) and C18:0 (0.80, p=1.63e-03), and a negative correlation with MUFA (-0.96, p=1.32e-06). The main contributions of FA to dimension 2 concern a positive correlation with n-6 PUFA (0.0.90, p=5.31e-05), C15:0 (0.89, p=1.09e-04), C22:6n-3 (0.79, p=1.97e-03) and a negative correlation with C20:5n-3 (-0.63, p=2.82e-02). Using the v.test from FactomineR, the statistical outputs performed on this PCA (Chi2) show nevertheless that the FA profile of plasma of the F1 Exposed female rabbits is significantly different of the control one (p=0.034), and that these differences are attributed to a positive association with C16:0 (v.test=2.057, p=0.039), and a negative association with C16:1n-7 (v.test=-2.426, p=0.015), C22:1n-9 (v.test=-2.502, p=0.012) and C18:3n-3 (v.test=-2.738, p=0.006), which confirm the statistical results obtained on data Table 2.

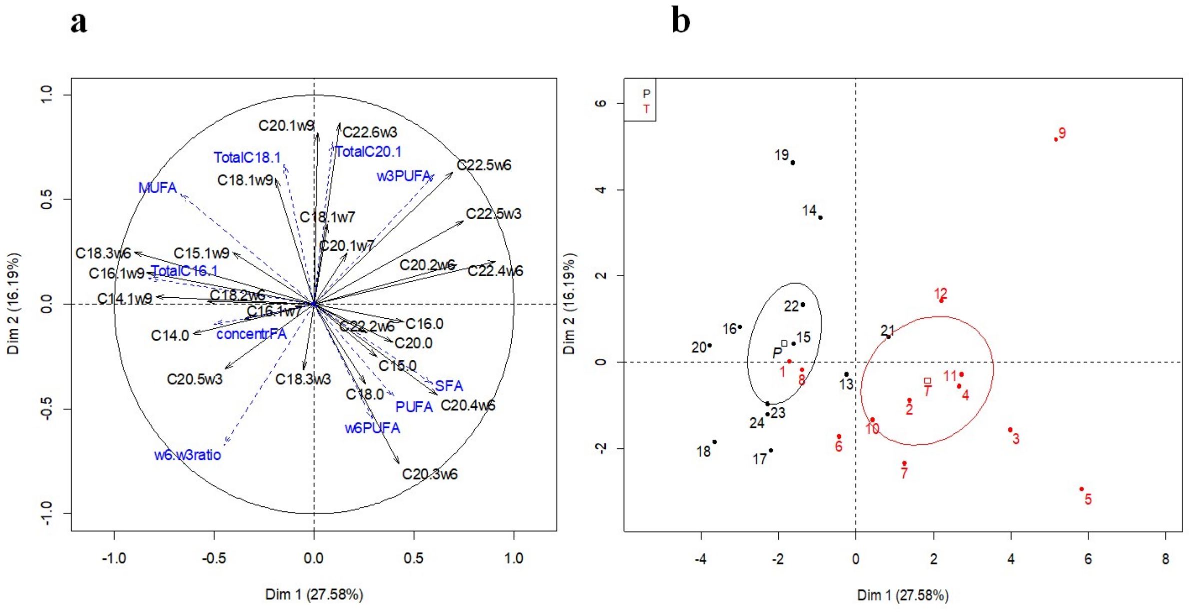


**Supplementary Figure S4 online: Principal Component Analyses (PCA) of the fatty acid profile of F2 placenta phospholipids (PL) at 28dpc.**

The PCA, plotting individual factor scores, provides a representation in a space of reduced dimensions, thus providing data structure and highlighting groups of homogeneous individuals. For this, we have looked for sub-areas in which projection of the cloud deforms as little as possible the initial data summarized in Table 3: Fatty acid profiles of F2 fetal plasma (with FA expressed as % of total FA). The first principal axis is the one that maximizes the variance when data are projected onto a line and the second one is orthogonal to it, and still maximizes the remaining variance. The variables, here the fatty acids of the placenta phospholipids profile, are plotted into a so-called Variables factor map (or "correlation circle" of the PCA, **A**), where the angle formed by any two variables represented as vectors, reflects their actual pairwise correlation. The Variables factor map allows identifying the variables that contribute "positively" vs. "negatively" to the PCA axes. The main plan, designed by the dimensions 1 and 2, represents 43.79% of the inertia of the data table, summarized in Table 3. The individual factor map (**B**) of this PCA shows that, as illustrated by the confidence ellipses drawn around the individuals, a specific FA signature characterized each group according to their *in utero* exposure, exposed (black) or control (red), with the dimension 1 that correlates with the *in utero* treatment of the F1 female (v.test =-3.37, R2= 0.49, p=0.0001). The main contribution of FA to dimension 1 concern a positive correlation (only the correlations >0.70 are reported here) with C22:4n-6 (0.91, p=9.22e-10), C22:5n-3 (0.75, p=2.83e-05), C22:2n-6 (0.72, p=8.51e-05), and a negative correlation with MUFA (-0.67, p=3.51e-04) and C14:0 (-0.61, p=1.62e-03). The main contribution of FA to dimension 2 concern a positive correlation with C22:6n-3(0.87, p=3.35e-08), C20:1n-9(0.82, p=8.54e-07), total C20:1(0.78, p=7.94e-06), total C18:1(0.67, p=3.51e-04),and a negative correlation with C20:3n-6 (-0.77, p=1.31e-05) and n-6/n-3 ratio (-0.67, p=3.12e-04). Using the v.test from FactomineR, the statistical outputs performed on this PCA (Chi2) confirm that the FA profile of placental phospholipids of the F1 Exposed female rabbits is highly significantly different of the control one with a sex interaction (TS, p=2.50e-05), and that these differences are attributed to a positive association with C18:3n-6 (v.test=3.07, p=0.0021), C16:1n-9 (v.test=2.59, p=0.0095), C18:2n-6 (v.test=2.32, p=0.0204), C14:1n-9 (v.test=2.16, p=0.0303), and a negative association with C22:4n-6 (v.test=-3.61, p=0.0003), C15:0(v.test=-3.03, p=0.0024), C22:5n-3 (v.test=-2.95, p=0.0032), C20:4n-6 (v.test=-2.76 p=0.0058), C20:2n-6 (v.test=-2.48, p=0.0131) and C22:5n-6 (v.test=-2.05, p=0.0406).

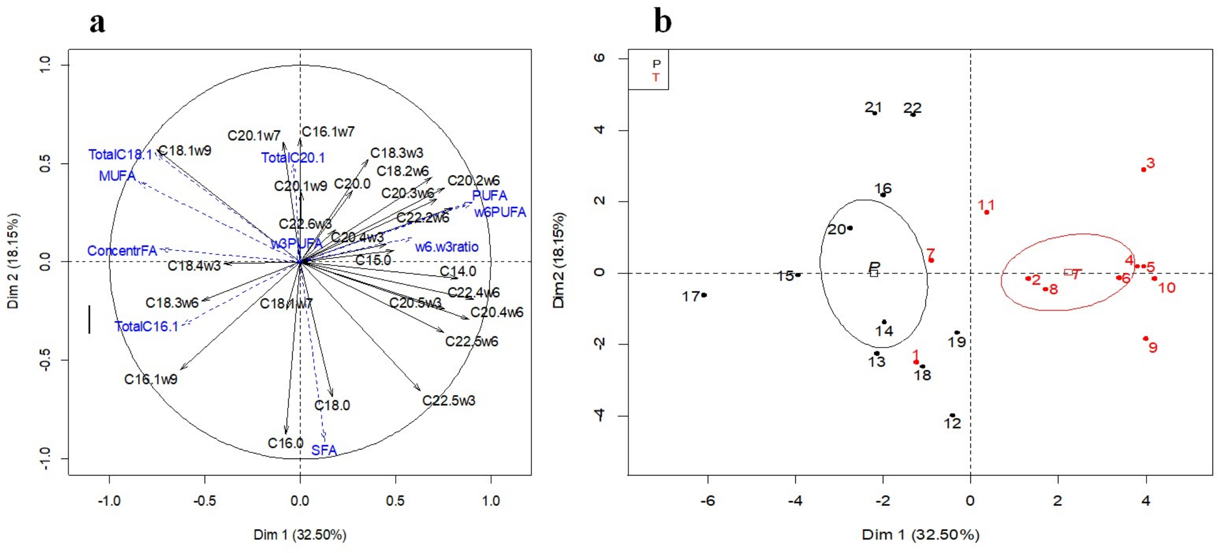


## Supplementary Figure S5 online: Principal Component Analyses (PCA) of the fatty acid profile of F2 placenta neutral lipids (NL) at 28dpc.

The PCA, plotting individual factor scores, provides a representation in a space of reduced dimensions, thus providing data structure and highlighting groups of homogeneous individuals. For this, we have looked for sub-areas in which projection of the cloud deforms as little as possible the initial data summarized in Table 4: Fatty acid profiles of F2 fetal plasma (with FA expressed as % of total FA). The first principal axis is the one that maximizes the variance when data are projected onto a line and the second one is orthogonal to it, and still maximizes the remaining variance. The variables, here the fatty acids of the placenta neutral lipid profile, are plotted into a so-called Variables factor map (or “correlation circle” of the PCA, **A**), where the angle formed by any two variables represented as vectors, reflects their actual pairwise correlation. The Variables factor map allows identifying the variables that contribute "positively" vs. "negatively" to the PCA axes. The main plan, designed by the dimensions 1 and 2, represents 50.65% of the inertia of the data table, summarized in Table 4. The individual factor map (**B**) of this PCA shows that, as illustrated by the confidence ellipses drawn around the individuals, a specific FA signature characterized each group according to their *in utero* exposure, exposed (black) or control (red), with the dimension 1 that correlates with the *in utero* treatment of the F1 female (v.test =-3.57, R2= 0.61, p=2.00e-05). The main contribution of FA to dimension 1 concern a positive correlation (only the correlations >0.80 are reported here) with C22:4n-6 (0.91 p=5.28e-09), n-6PUFA (0.90, p=1.20e-08), PUFA (0.88, p=4.58e-08), C20:4n-6 (0.88, p=5.56e-08), C14:0 (0.83, p=2.04e-06), and a negative correlation with MUFA (-0.85, p=7.13e-07), total C18:1(-0.76, p=4.04e-05). The main contribution of FA to dimension 2 concern a positive correlation with C16:1n-7(0.63, p=1.61e-03), C20:1n-7(0.61, p=2.55e-03), C18:1n-9(0.57, p=5.18e-03), and a negative correlation with SFA (-0.91, p=5.71e-09), C16:0 (-0.87, p=1.23e-07), and C18:0(-0.68, p=4.49e-04). Using the v.test from FactomineR, the statistical outputs performed on this PCA (Chi2) confirm that the FA profile of placental NL from the F1 Exposed female rabbits is highly significantly different of the control one with a sex interaction (TS, with exposed male > exposed females, p=5.52e-05), and that these differences are attributed to a positive association with C18:1n-9 (v.test=3.08, p=0.0021), C16:1n-9 (v.test=2.04, p=0.0416), and a negative association with C14:0 (v.test=-3.58, p=0.0003), C20:4n-6(v.test=-3.35, p=0.0008), C18:2n-6 (v.test=-3.12, p=0.0018), C22:4n-6 (v.test=-3.07 p=0.0021), C20:5n-3 (v.test=-2.99, p=0.0028), C15:0 (v.test=-2.75, p=0.0059), C18:3n-3 (v.test=-2.28, p=0.0222), and C20:3n-6 (v.test=-2.16, p=0.0305).

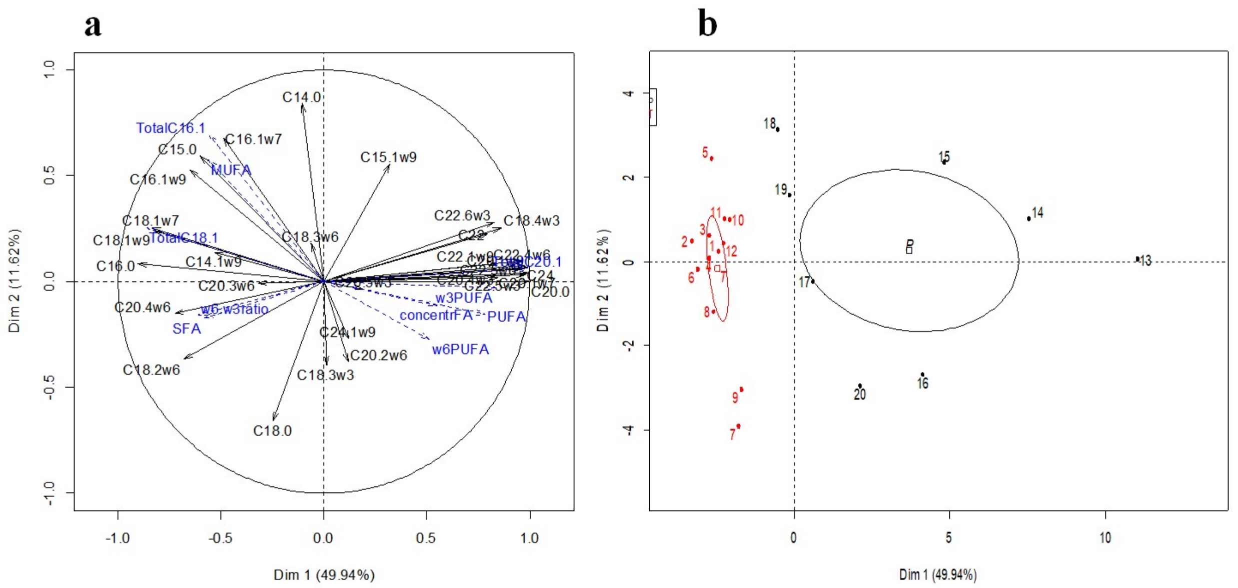


**Supplementary Figure S6 online: Principal Component Analyses (PCA) of the fatty acid profile of plasma of F2 fetus at 28dpc.**

The PCA, plotting individual factor scores, provides a representation in a space of reduced dimensions, thus providing data structure and highlighting groups of homogeneous individuals. For this, we have looked for sub-areas in which projection of the cloud deforms as little as possible the initial data summarized in Table 5: Fatty acid profiles of F2 fetal plasma (with FA expressed as % of total FA). The first principal axis is the one that maximizes the variance when data are projected onto a line and the second one is orthogonal to it, and still maximizes the remaining variance. The variables, here the fatty acids of the plasma profile, are plotted into a so-called Variables factor map (or "correlation circle" of the PCA, **a**), where the angle formed by any two variables represented as vectors, reflects their actual pairwise correlation. The Variables factor map allows identifying the variables that contribute "positively" vs. "negatively" to the PCA axes. The main plan, designed by the dimensions 1 and 2, represents 61.56% of the inertia of the data table, summarized in Table 6. The individual factor map (**b**) of this PCA shows that, as illustrated by the confidence ellipses drawn around the individuals, a specific FA signature characterized each group according to their *in utero* exposure, exposed (black) or control (red), with the dimension 1 that correlates with the *in utero* treatment of the F1 female (v.test =3.39, R2= 060, p=5.05e-05). The main contribution of FA to dimension 1 concern a positive correlation (only the correlations >0.80 are reported here) with C20:0 (0.99 p=2.73e-19) , C20:1n-7(0.99, p=5.16e-17), C20:1n-9 (0.99, p=2.30e-16), C22:5n-3(0.97, p=3.40e-03), C24:0 (0.97, p=5.95e-13), C22:4n-6:0 (0.96, p=8.72e-12), C22:5n-6 (0.95, p=9.90e-11), C28:4n-3 (0.86, p=1.18e-06), and a negative correlation with C18:1n-7 (-0.84, p=4.32e-06), C18:1n-9(-0.82, p=7.67e-06), and C20:4n-6 (-0.72, p=3.18e-04). The main contributions of FA to dimension 2 concern a positive correlation with C14:0(0.84, p=3.35e-06) and a negative correlation with C18:0 (-0.66, p=1.71e-03). Using the v.test from FactomineR, the statistical outputs performed on this PCA (Chi2) confirm that the FA profile of plasma of the F1 Exposed female rabbits is highly significantly different of the control one (p=0.006), and that these differences are attributed to a positive association with C20:1n-7 (v.test=3.65, p=0.0003), C20:1n-9 (v.test=3.52, p=0.0004), C20:0 (v.test=3.48, p=0.0005), C22:0 (v.test=3.42, p=0.0006), C22:5n-3 (v.test=3.22, p=0.0012) , C22:4n-6 (v.test=3.13, p=0.0017), and C22:1n-9 (v.test=3.02, p=0.0025), and a negative association with C20:4n-6 (v.test=-3.74, p=0.0018), C18:1n-7(v.test=-3.59, p=0.0003), C20:3n-6 (v.test=-2.96, p=0.0031) and C16:0 (v.test=-2.91, p=0.0036).

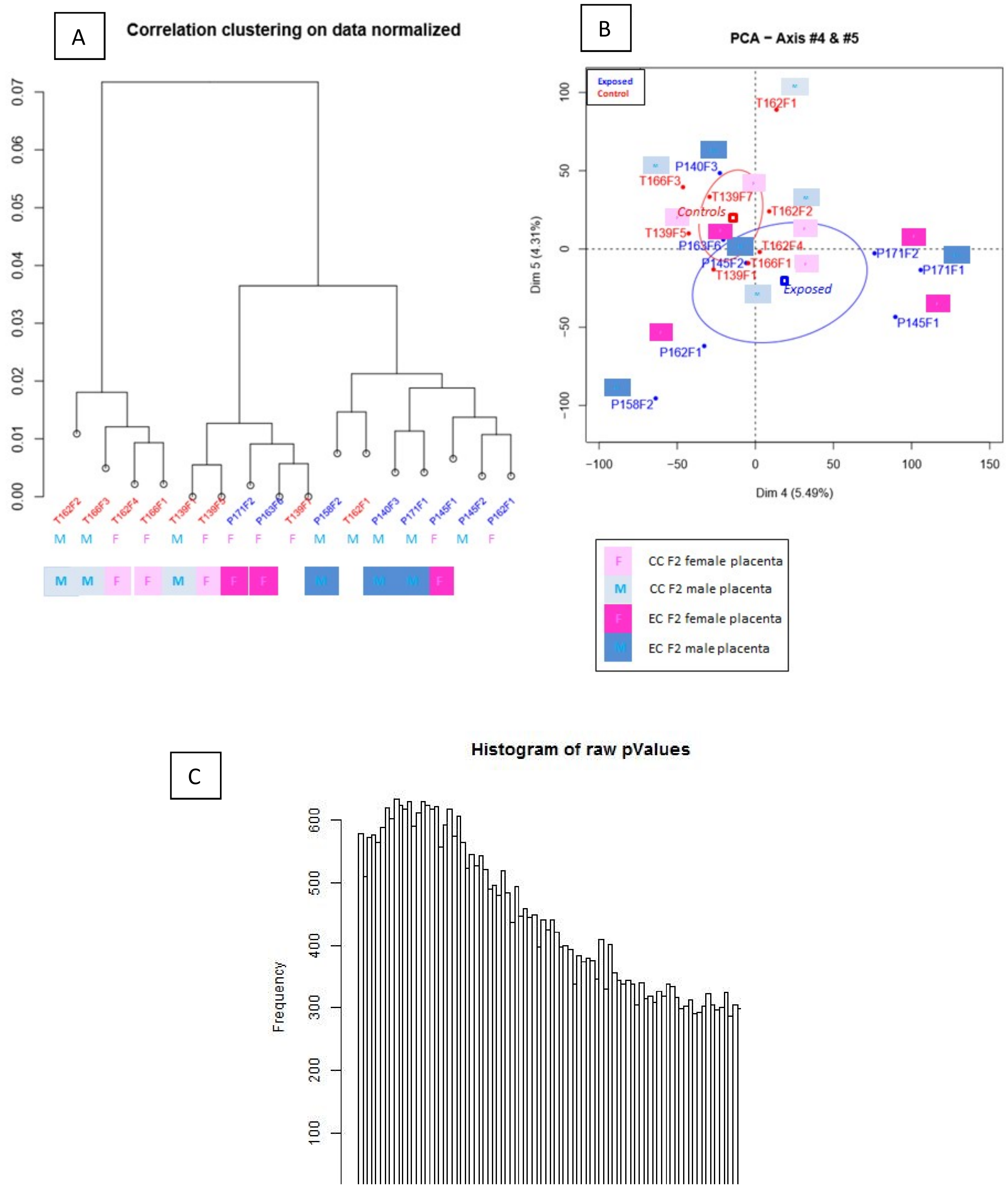


**Supplemental Figure S7 online. Hierarchical clustering of transcriptomes of F2 EC (noted P in blue on the graphs) and CC (noted T in red on the graphs) placentas (n=8 per condition), in rabbit.**

A. Hierarchical clustering was based on the Pearson correlation coefficient of F2 EC and CC placentas transcriptome. B. Correlation circles around the groups of the Principal Component Analyses (PCA) in the plane constituted by the dimensions 4 and 5 presented here for example. Foetal sex among groups is indicated with different colours. C. Histogram of raw pValues (justifying the use of GSEA analyses).

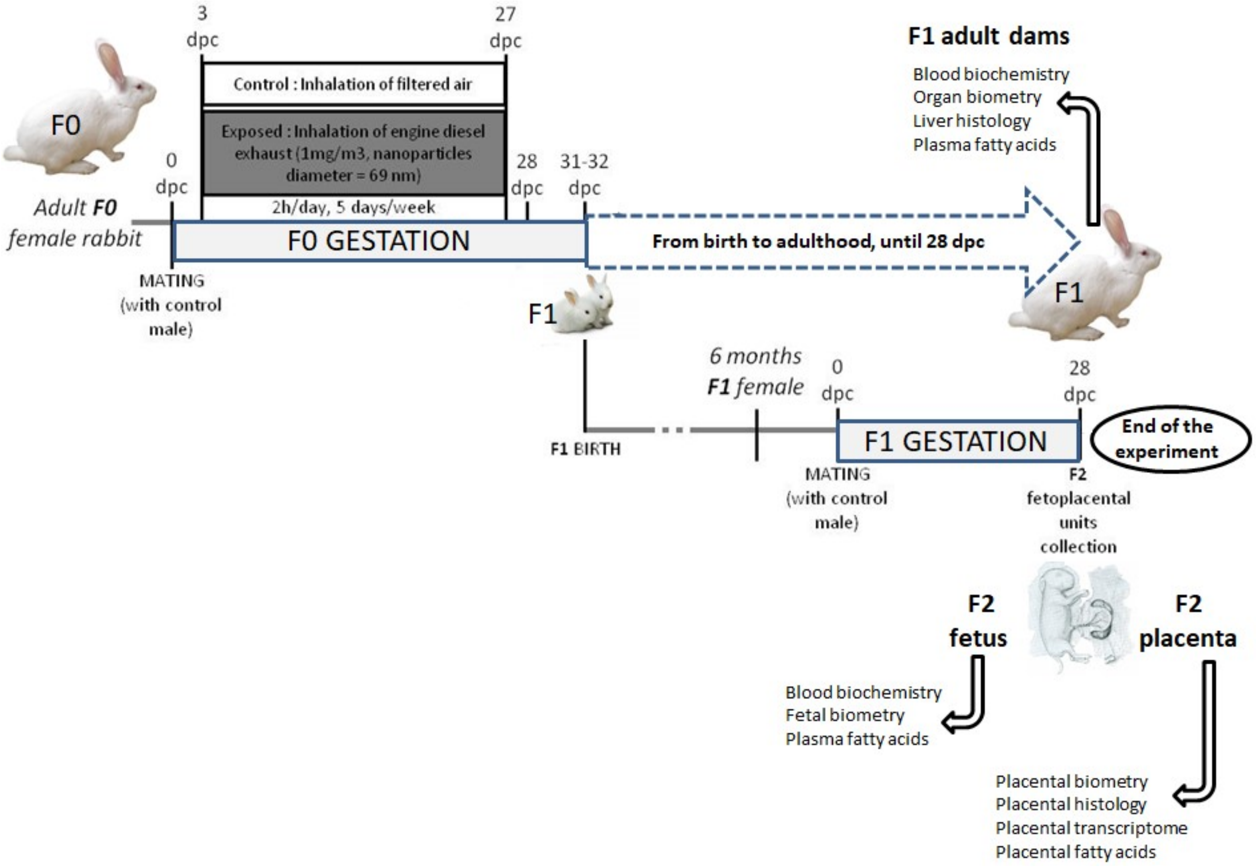


**Supplemental Figure S8 online. Experimental protocol from initial exposure of F0 females to diesel exhaust to collection of F2 feto-placental units, in rabbit (modified from Valentino et al., PFT 2016, doi: 10.1186/s12989-016-0151-7).**

*Sixteen F0 females (N=7 Controls, N=9 Exposed dams) gave birth to F1 offspring, which were raised under control conditions. Altogether, 72 F1 offspring survived until adulthood, including 18 control females, 40 exposed females, 11 control males and 16 exposed males. All animals were weaned at 5 weeks of age. After becoming sexually mature adults at 6.5 months of age, a limited number of F1 nulliparous females (11 F1 control and 11 F1 exposed females) were dedicated to the production of the F2 generation. These females were mated with unexposed males. In the controls, only 5 out 11 rabbits were diagnosed as pregnant, among which one rabbit died at mid-pregnancy and another aborted; in the exposed group 10 out 11 rabbits were pregnant, including 1 abortion at mid-pregnancy. All females that were still pregnant (N=3 controls and N=9 exposed) were euthanized at 28 dpc to collect second generation (F2) foeto-placental units. The litter sizes were 6 to 13 fetuses without difference in sex ratio or change in fetal weight between groups.*

## Supplementary Tables online

### Supplementary Table S1 online: Maternal biochemistry at 28dpc (control (C) or *in utero* exposed (E) F1 pregnant dams)

| Variable | Number o dams | | Median [Q1; Q3] | | Fully adjusted linear model | | |
|---|---|---|---|---|---|---|---|
| | C | E | C | E | β value | CI | p-value |
| Glycemia (mmol/L) | 3 | 9 | 6.560 [6.020; 6.900] | 5.630 [5.090; 7.070] | -0.274 | [-2.145; 1.598] | 0.786 |
| Insulin (mUI/L) | 3 | 9 | 4.300 [4.190; 9.610] | 2.430 [1.280; 4.650] | -2.606 | [-7.367; 2.155] | 0.339 |
| Total cholesterol (mmol/L) | 3 | 9 | 0.200 [0.170; 0.210] | 0.190 [0.165; 0.190] | -0.004 | [-0.041; 0.032] | 0.820 |
| Triglycerides (mmol/L) | 3 | 9 | 0.190 [0.170; 0.220] | 0.240 [0.210; 0.265] | 0.157 | [-0.201; 0.515] | 0.434 |
| ASAT (UI/L) | 3 | 9 | 29.00 [23.00; 29.00] | 35.50 [28.50; 42.00] | 8.125 | [-0.363; 16.61] | 0.131 |
| ALAT (UI/L) | 3 | 9 | 18.00 [15.00; 20.00] | 12.00 [7.500; 18.50] | -4.778 | [-11.71; 2.151] | 0.244 |
| Creatinine (µmol/L) | 3 | 9 | 84.00 [83.00; 111.0] | 102.0 [81.00; 109.0] | -0.095 | [-27.37; 27.18] | 0.995 |
| Urea (mmol/L) | 3 | 9 | 4.900 [4.200; 5.100] | 4.600 [3.650; 5.350] | 0.122 | [-1.627; 1.871] | 0.897 |

ASAT: Aspartate Aminotransferase; ALAT: Alanine Aminotransferase. F0 female rabbits inhaled 1mg/m$^3$ of NPs, 2 hours/day, 5 days/week, from 3 dpc to 27dpc during gestation. Dams were allowed to give birth to the generation F1. Adult F1 female (7.5 months of age), *in utero* exposed, were mated and euthanized when pregnant at 28 dpc.

### Supplementary Table S2 online: Stereological data of placenta for the second generation

| Variable | Number of fetus | | Median [Q1; Q3] | | Fully adjusted linear model | | |
|---|---|---|---|---|---|---|---|
| | CC | EC | CC | EC | β value | CI | p-value |
| Volume fraction of trophoblast | 6 | 6 | 33.66 [28.45; 38.50] | 35.20 [31.97; 41.71] | 4.075 | [-8.041; 16.19] | 0.542 |
| Volume fraction of fetal capillaries | 6 | 6 | 22.01 [17.70; 25.79] | 16.95 [12.78; 26.48] | -3.609 | [-10.87; 3.649] | 0.381 |
| Volume fraction of maternal blood space | 6 | 6 | 38.33 [31.71; 46.27] | 38.48 [33.75; 43.00] | 0.105 | [-8.199; 8.408] | 0.981 |
| Surface density of trophoblast | 6 | 6 | 0.252 [0.203; 0.278] | 0.241 [0.179; 0.281] | -0.008 | [-0.047; 0.030] | 0.692 |
| Surface density of fetal capillaries | 6 | 6 | 0.248 [0.201; 0.296] | 0.220 [0.188; 0.251] | -0.027 | [-0.075; 0.021] | 0.328 |
| Surface density of maternal blood space | 6 | 6 | 0.269 [0.244; 0.320] | 0.245 [0.211; 0.276] | -0.047 | [0.120; 0.026] | 0.269 |

F0 female rabbits inhaled 1mg/m3 of NPs, 2 hours/day, 5 days/week, from 3 dpc to 27dpc during gestation. Dams were allowed to give birth to the generation F1. Adult F1 female (7.5 months of age), in utero exposed, were mated and euthanized when pregnant at 28 dpc. Fetoplacental units of generation F2 were collected in control (CC) and exposed (EC) groups. Placental labyrinthine was fixed in Formalin and included in paraffin for histological analysis. After preparation of histological sections with microtome, immunodetection of vimentin to label fetal capillaries was performed on labyrinthine area sections from the control group and the exposed group. Volume fraction of trophoblast, fetal capillaries and maternal blood space and surface density of fetal capillaries were quantified. Effect of grand-dam (F0) pregnancy exposure to engine diesel exhaust on second-generation fetuses was estimated using linear model with random effect of dam (F1) adjusted for number of fetuses by dam, fetus position in the horn and fetus sex. All data are expressed as median[Q1;Q3].(*p < 0.05).

**Supplementary Table S3 online: Differential gene expression in transcriptomics analyses in EC versus CC placentas, normalized signal and annotated genes** (cf. file in .txt format)

**Supplementary Table S4 online: Gene set Enrichment Analysis - Database C2:KEGG.**

| Population | Pathway | Annoted gene number | Enrichment Core gene number | NES | FDR q-value |
|---|---|---|---|---|---|
| Exposed | RNA degradation | 41 | 22 | 2.07 | 0.015 |
| | Ubiquitin mediated proteolysis | 98 | 40 | 1.96 | 0.033 |
| | Protein export | 16 | 12 | 1.93 | 0.024 |
| | Aminoacyl TRNA biosynthesis | 35 | 20 | 1.86 | 0.035 |
| | N-Glycan biosynthesis | 29 | 14 | 1.79 | 0.049 |
| | Adherens Junction | 62 | 19 | 1.77 | 0.049 |
| | Endocytosis | 125 | 49 | 1.71 | 0.066 |
| | Sphingolipid metabolism | 27 | 11 | 1.69 | 0.069 |
| | Valine/Leucine/Isoleucine degradation | 37 | 18 | 1.52 | 0.209 |
| | Chronic myeloid Leukemia | 59 | 25 | 1.5 | 0.215 |
| Control | Olfactory transduction | 82 | 56 | -2.03 | 0.000 |
| | Neuroactive ligand receptor interaction | 181 | 127 | -2.02 | 0.000 |
| | Systemic lupus erythematosus | 40 | 27 | -1.87 | 0.009 |
| | Allograft rejection | 18 | 11 | -1.78 | 0.023 |
| | Autoimmune Thyroid disease | 19 | 12 | -1.75 | 0.029 |
| | Graft versus hist disease | 15 | 11 | -1.73 | 0.030 |
| | Intestinal immune network for IGA production | 30 | 21 | -1.67 | 0.059 |
| | Drug metabolism other enzymes | 23 | 8 | -1.66 | 0.061 |
| | Dilated cardiomyopathy | 71 | 27 | -1.54 | 0.163 |
| | Cardiac muscle contraction | 43 | 17 | -1.53 | 0.170 |
| | Calcium signaling pathway | 130 | 58 | -1.50 | 0.197 |
| | Complement and coagulation cascades | 52 | 35 | -1.50 | 0.186 |
| | Primary immunodeficiency | 26 | 12 | -1.49 | 0.180 |
| | Hypertrophic cardiomyopathy HCM | 68 | 26 | -1.47 | 0.209 |
| | Cell adhesion molecules cams | 92 | 43 | -1.43 | 0.254 |
| | Adipocytokine signaling pathway | 58 | 21 | -1.43 | 0.247 |

FDR: False Discovery Rate; NES: Normalized Enrichment Score.

*Gene set enrichment analysis (GSEA) (also known as functional enrichment analysis) is a method to identify classes of genes that are over-represented in a large set of genes, and may have an association with disease phenotypes or biological processes. The method uses statistical approaches to identify significantly enriched or depleted groups of genes. Microarray results often identify thousands of genes which are used for the analysis. Gene set enrichment analysis uses a priori gene sets that have been grouped together by their involvement in the same biological pathway. The top and bottom of the list correspond to the largest differences in expression between the two cell types or conditions. If the gene set falls at either the top (over-expressed) or bottom (under-expressed), it is thought to be related to the phenotypic differences. In the method that is typically referred to as standard GSEA, there are three steps involved in the analytical process. The general steps are summarized below: 1) Calculate the enrichment score (ES) that represents the amount to which the genes in the set are over-represented at either the top or bottom of the list. This score is a Kolmogorov–Smirnov-like statistic. 2) Estimate the statistical significance of the ES. This calculation is done by a phenotypic-based permutation test in order to produce a null distribution for the ES. 3) Adjust for multiple hypothesis testing for when a large number of gene sets are being analyzed at one time. The enrichment scores for each set are normalized and a false discovery rate is calculated.*

**Supplementary Table S5 online: Gene set Enrichment Analysis - Database Gene Ontology, C5:BP.**

| Population | Pathway | Annoted gene number | Enrichment Core gene number | NES | FDR q-value |
|---|---|---|---|---|---|
| Exposed | Golgi vesicle transport | 33 | 16 | 2.22 | 0.012 |
| | Phosphoinositide metabolic process | 21 | 9 | 1.93 | 0.103 |
| | Intracellular transport | 210 | 54 | 1.89 | 0.094 |
| | Endosome transport | 18 | 7 | 1.87 | 0.085 |
| | Ubiquitin cycle | 31 | 14 | 1.82 | 0.096 |
| | Protein ubiquitination | 24 | 12 | 1.81 | 0.085 |
| | Viral infectious cycle | 22 | 11 | 1.75 | 0.122 |
| | Intracellular protein transport | 114 | 39 | 1.73 | 0.125 |
| | Protein modification by small protein conjugation | 26 | 13 | 1.68 | 0.158 |
| | Phosphoinositide biosynthetic process | 15 | 6 | 1.66 | 0.159 |
| | Sphingolipid metabolic process | 22 | 7 | 1.65 | 0.154 |
| | Protein transport | 122 | 40 | 1.58 | 0.222 |
| | Membrane lipid metabolic process | 72 | 21 | 1.56 | 0.248 |
| | Establishment of cellular localization | 256 | 58 | 1.56 | 0.231 |
| Control | Oxygen and reactive oxygen species matebolic process | 18 | 7 | -1.78 | 0.219 |
| | Development of primary sexual characteristics | 19 | 6 | -1.68 | 0.243 |
| | Detection of stimulus | 34 | 22 | -1.66 | 0.236 |
| | Excretion | 26 | 13 | -1.66 | 0.214 |
| | Regulation of secretion | 27 | 16 | -1.65 | 0.199 |
| | Negative regulation of protein metabolic process | 35 | 14 | -1.65 | 0.197 |
| | Second messenger mediated signaling | 106 | 62 | -1.63 | 0.202 |
| | Negative regulation of protein metabolic process | 33 | 13 | -1.62 | 0.211 |
| | Metal ion transport | 85 | 51 | -1.62 | 0.202 |
| | Monovalent inorganic cation transport | 64 | 39 | -1.62 | 0.190 |
| | Brain development | 37 | 22 | -1.61 | 0.196 |
| | System process | 380 | 179 | -1.61 | 0.180 |
| | Neurological system process | 259 | 143 | -1.59 | 0.207 |
| | G protein signaling coupled to cyclic nucleotide second messenger | 69 | 41 | -1.58 | 0.218 |
| | Transmission of nerve impulse | 125 | 64 | -1.57 | 0.230 |
| | Cyclic nucleotide mediated signaling | 71 | - | -1.56 | 0.242 |
| | Lymphocyte activation | 41 | - | -1.55 | 0.243 |
| | Cellular defense response | 31 | - | -1.55 | 0.242 |

FDR: False Discovery Rate; NES: Normalized Enrichment Score.

**Supplementary Table S6 online: Gene set Enrichment Analysis - Database Gene Ontology, C5:CC.**

| Population | Pathway | Annoted gene number | Enrichment Core gene number | NES | FDR q-value |
|---|---|---|---|---|---|
| Exposed | Proteasome complex | 18 | 12 | 2.16 | 0.016 |
| | Nuclear pore | 21 | 15 | 2.07 | 0.013 |
| | Nuclear envelope | 50 | 24 | 2.03 | 0.020 |
| | Integral to organelle membrane | 42 | 19 | 2.02 | 0.016 |
| | Organelle membrane | 207 | 88 | 1.96 | 0.019 |
| | Nuclear membrane part | 28 | 17 | 1.95 | 0.018 |
| | Endomembrane system | 158 | 72 | 1.92 | 0.020 |
| | Peroxisome | 35 | 14 | 1.90 | 0.021 |
| | Nulear membrane | 33 | 18 | 1.89 | 0.020 |
| | Intrinsic to organelle membrane | 44 | 19 | 1.86 | 0.021 |
| | Microbody | 35 | 14 | 1.86 | 0.020 |
| | Pore complex | 25 | 15 | 1.84 | 0.021 |
| | Golgi membrane | 38 | 21 | 1.76 | 0.039 |
| | Organelle envelope | 110 | 34 | 1.66 | 0.067 |
| | Envelope | 110 | 34 | 1.65 | 0.066 |
| | Endoplasmic reticulum | 194 | 60 | 1.62 | 0.075 |
| | DNA directed RNA polymerase II holoenzyme | 42 | 15 | 1.56 | 0.106 |
| | Nuclear part | 388 | 116 | 1.54 | 0.114 |
| | Golgi Appartus part | 83 | 36 | 1.52 | 0.124 |
| Control | Extracellular matrix | 79 | 31 | -1.72 | 0.185 |
| | Proteinaceous extracellular matrix | 78 | 31 | -1.71 | 0.097 |
| | Spindle | 30 | 11 | -1.66 | 0.121 |
| | Microtubule cytoskeleton | 114 | 31 | -1.66 | 0.093 |
| | Extracellular region part | 241 | 124 | -1.66 | 0.075 |
| | Collagen | 22 | 9 | -1.60 | 0.101 |
| | Voltage gated potassium channel complex | 23 | 13 | -1.55 | 0.145 |
| | Extracellular region | 313 | 155 | -1.53 | 0.145 |
| | Extracellular space | 167 | 99 | -1.53 | 0.134 |
| | Cytoskeleton | 271 | 66 | -1.46 | 0.199 |
| | Cytoskeletal part | 179 | 38 | -1.45 | 0.208 |

FDR: False Discovery Rate; NES: Normalized Enrichment Score.

**Supplementary Table S7 online: Gene set Enrichment Analysis - Database Gene Ontology, C5:MF.**

| Population | Pathway | Annoted gene number | Enrichment Core gene number | NES | FDR q-value |
|---|---|---|---|---|---|
| Exposed | Signal sequence binding | 15 | 6 | 2.24 | 0.007 |
| | Small protein conjugating enzyme activity | 35 | 15 | 2.13 | 0.013 |
| | Ubiquitin protein ligase activity | 34 | 14 | 2.08 | 0.014 |
| | Ligase activity forming carbon nitrogen bonds | 48 | 17 | 2.08 | 0.013 |
| | Cysteine type peptidase activity | 43 | 15 | 2.06 | 0.011 |
| | Acid/amino acid ligase activity | 40 | 15 | 1.99 | 0.016 |
| | Small conjugating protein ligase activity | 36 | 14 | 1.98 | 0.016 |
| | Cysteine type endopeptidase activity | 35 | 14 | 1.76 | 0.077 |
| | Ligase activity | 72 | 23 | 1.65 | 0.139 |
| | Hydrolase activity acting on acid anhydrides | 170 | 66 | 1.65 | 0.130 |
| | Coenzyme binding | 15 | 9 | 1.64 | 0.120 |
| | Pyrophosphatase activity | 168 | 66 | 1.64 | 0.114 |
| | Nucleoside triphosphate activity | 157 | 62 | 1.61 | 0.129 |
| | GTPase activity | 62 | 24 | 1.57 | 0.156 |
| | RNA helicase activity | 16 | 8 | 1.52 | 0.188 |
| Control | Rhodopsin like receptor activity | 80 | 54 | -1.93 | 0.026 |
| | Amine receptor activity | 22 | 16 | -1.83 | 0.067 |
| | Cation channel activity | 81 | 50 | -1.80 | 0.063 |
| | G protein coupled receptor activity | 121 | 77 | -1.77 | 0.067 |
| | G protein coupled receptor binding | 31 | 20 | -1.75 | 0.066 |
| | Substrate specific channel activity | 108 | 71 | -1.73 | 0.071 |
| | Ion channel activity | 103 | 66 | -1.72 | 0.063 |
| | Chemokine activity | 21 | 12 | -1.62 | 0.156 |
| | Chemokine receptor binding | 21 | 12 | -1.61 | 0.151 |
| | Calcium channel activity | 24 | 14 | -1.61 | 0.148 |
| | Gated channel activity | 86 | 53 | -1.59 | 0.164 |
| | Cation transmembrane transporter activity | 154 | 53 | -1.58 | 0.162 |
| | Voltage gated cation channel activity | 45 | 31 | -1.57 | 0.163 |
| | Voltage gated channel activity | 49 | 33 | -1.55 | 0.185 |
| | Cytokine activity | 66 | 32 | -1.54 | 0.191 |
| | Ion transmembrane transporter activity | 203 | 64 | -1.53 | 0.195 |
| | Transmembrane receptor activity | 277 | 137 | -1.51 | 0.211 |
| | Hormone activity | 31 | 14 | -1.50 | 0.211 |
| | Pattern binding | 29 | 19 | -1.49 | 0.214 |
| | Metal ion transmembrane transporter activity | 104 | 62 | -1.48 | 0.221 |
| | Protein C terminus binding | 50 | - | -1.48 | 0.225 |
| | Extracellular matrix structural constituent | 20 | - | -1.47 | 0.225 |
| | Receptor binding | 266 | - | -1.46 | 0.225 |
| | Motor activity | 21 | - | -1.46 | 0.217 |

FDR: False Discovery Rate; NES: Normalized Enrichment Score.
